# Understanding Computational Costs of Cellular-Level Brain Tissue Simulations Through Analytical Performance Models

**DOI:** 10.1007/s12021-019-09451-w

**Published:** 2020-02-13

**Authors:** Francesco Cremonesi, Felix Schürmann

**Affiliations:** Blue Brain Project, Brain Mind Institute, École polytechnique fédérale de Lausanne (EPFL), Campus Biotech, 1202 Geneva, Switzerland

**Keywords:** Computational models of neurons, Brain tissue simulations, Performance modeling, High performance computing

## Abstract

Computational modeling and simulation have become essential tools in the quest to better understand the brain’s makeup and to decipher the causal interrelations of its components. The breadth of biochemical and biophysical processes and structures in the brain has led to the development of a large variety of model abstractions and specialized tools, often times requiring high performance computing resources for their timely execution. What has been missing so far was an in-depth analysis of the complexity of the computational kernels, hindering a systematic approach to identifying bottlenecks of algorithms and hardware. If whole brain models are to be achieved on emerging computer generations, models and simulation engines will have to be carefully co-designed for the intrinsic hardware tradeoffs. For the first time, we present a systematic exploration based on analytic performance modeling. We base our analysis on three *in silico* models, chosen as representative examples of the most widely employed modeling abstractions: current-based point neurons, conductance-based point neurons and conductance-based detailed neurons. We identify that the synaptic modeling formalism, i.e. current or conductance-based representation, and not the level of morphological detail, is the most significant factor in determining the properties of memory bandwidth saturation and shared-memory scaling of *in silico* models. Even though general purpose computing has, until now, largely been able to deliver high performance, we find that for all types of abstractions, network latency and memory bandwidth will become severe bottlenecks as the number of neurons to be simulated grows. By adapting and extending a performance modeling approach, we deliver a first characterization of the performance landscape of brain tissue simulations, allowing us to pinpoint current bottlenecks for state-of-the-art *in silico* models, and make projections for future hardware and software requirements.

**Electronic supplementary material:**

The online version of this article (10.1007/s12021-019-09451-w) contains supplementary material, which is available to authorized users.

## Introduction

In the field of computational neuroscience, simulations of biological neural networks represent one of the fundamental tools for hypothesis testing and exploration. A widely used scale of representation are neuron-based approaches, i.e. models of brain tissue in which the fundamental unit is represented by a neuronal cell. This representation is important as it allows for a faithful matching of the model with a range of anatomical and electrophysiological data (Markram et al. 2015; Potjans and Diesmann 2012; Pozzorini et al. 2015; Hagen et al. 2016, 2018). While determining the adequate level of detail is a formidable challenge with respect to the system modeled, so is the addressing of an efficient implementation of the simulation in software. The size of such networks, both in terms of number of neurons and synapses and rate of synaptic events, as well as the level of biological detail required to answer meaningful questions about the brain, mean that these simulations come at a large computational cost.

Much of the early increase in computational requirements of models and simulations have been supported by Dennard scaling (1974) and Moore’s law (1995), but with chip-manufacturing technology reaching its limit and the consequent rise of multi-core and heterogeneous architectures (Hardavellas et al. 2011; Simonite 2016), computational neuroscientists have been forced to develop more efficient algorithms and software to be able to keep up with the increasing demands of modellers. Research efforts in the context of simulation neuroscience have investigated the efficient utilization of modern multicore processors (see e.g. Kumbhar et al. 2016, 2018; Eichner et al. 2009; Brette and Goodman 2011), parallel computing (see e.g. Morrison et al. 2005; Ovcharenko et al. 2015; Helias et al. 2012), accelerators (see e.g. Knight and Nowotny 2018; Fidjeland et al. 2009; Brette and Goodman 2012) and brain-inspired hardware (see e.g. Painkras et al. 2013; Benjamin et al. 2014; Indiveri et al. 2011).

Despite the multiple years of research in efficient implementations of neuron models, we are still missing a more quantitative treatment of what are the actual computational characteristics of a given level of detail and how a particular level of detail may be limited by specific hardware trade-offs. How much more costly is a morphologically detailed neuron simulation compared to a representation modeling the same neuron as a point? What is the influence of how the synapses are being modeled? Can we expect that point neuron models can scale to massively parallel computers in a similar way than detailed neuron models?

For the first time, we extend performance modeling techniques to the field of computational neuroscience, allowing us to establish a quantitative relationship between the parameters dictated by the biophysical model, the complexity properties of the simulation algorithm and the details of the hardware specifications. Although we require a reasonable level of accuracy and validation against benchmarks, our goal is not to obtain highly accurate performance predictions, but rather to design a tool with sufficient generality to identify current and future bottlenecks for different levels of abstraction on the spectrum of models for neural cells’ dynamics. Based on our requirements, we choose a performance modeling approach that is neither purely based on first-principles (see e.g. Williams et al. 2009) nor purely empirical (see e.g. Calotoiu et al. 2013) but is instead an hybrid approach known as grey-box analytical modeling. Our analysis is based on state-of-the-art high performance computing (HPC) hardware architecture and applied to three published neural network simulations that have been selected to represent the diversity of neuron models in the literature.

Our analysis shows that there are significant differences in the performance profiles of *in silico* models falling within the same category of cell-based representations. Features related not only to the neuron abstraction but also to the scientific question under analysis can cause variations in the hardware bottlenecks and ultimately influence simulation performance. We find instances where the level of morphological detail is not a factor in distinguishing between models’ performance, while the synaptic formalism is, allowing us to identify the key factors that determine a model’s computational profile. Finally, we show that our analysis is strongly conditioned on the published models’ parameters and simulation dynamics, predicting how changes in some values, notably the firing frequency, can significantly alter the performance profile. Short of being able to run benchmarks on different kinds of hardware architectures, we use our model to explore the effects of machine balance and hardware design choices on the performance of *in silico* brain tissue simulations, providing actionable guidelines for hardware procurement and co-design. Ultimately we come to the conclusion that while general-purpose computing has, until now, largely been able to deliver high performance, the next generation of brain tissue simulation will be severely limited by hardware bottlenecks. Indeed this trend was already foreshadowed in empirical studies (Jordan et al. 2018), and solutions involving hardware accelerators such as General Purpose Graphical Processing Units (GPGPUs) have been proposed (Fidjeland et al. 2009; Yavuz et al. 2016; Brette and Goodman 2012), while the development of custom brain-like hardware is being actively explored with promising results (van Albada et al. 2018; Wunderlich et al. 2018).

We believe that the situation calls for a better, deeper understanding of how hardware capabilities interact with brain tissue simulation algorithms. In turn, this would allow a stronger collaboration between *in silico* modelers, developers and hardware specialists to orchestrate the co-design of software and hardware architectures. The methodology developed in this work constitutes a quantitative means through which these scientific communities can collaborate in the task of designing and optimizing future software and hardware for the next generation of brain tissue simulations.

### Related Work

As a testament to the growing interest of the community in the performance of brain tissue simulations, several studies on this topic have been published in the literature. To our knowledge, however, none of them have used performance modeling as a tool to explain the empirically observed performance properties of simulations, nor have they tried to analyze such a wide scope of models as we do in this paper.

The review work of Brette et al. (2007) presented a large number of different simulators and corresponding *in silico* models, but included only basic formulas for asymptotic complexity, without exploring the complicated effect that implementation and hardware have on measured performance. In a series of papers, the developers and users of the NEST software have investigated the issues related to scaling simulations of neurons to very large scales (Kunkel et al. 2014; Peyser and Schenck 2015; Ippen et al. 2017; Jordan et al. 2018) and proposed solutions to avoid the performance bottlenecks they encountered. Similarly, new simulators and spike communication strategies have been explored (Ananthanarayanan and Modha 2007; Kozloski and Wagner 2011; Hines et al. 2011). All these studies provide very useful data to compare against our own model and conclusions, but are restricted to distributed simulations and focus on optimizing efficiency using novel algorithmic and implementation techniques. A performance model for a NEST simulation was fully described in Schenck et al. (2014), but focuses only on distributed simulations, neglecting single-node performance, and uses a different performance modeling approach based on interpolating empirical observations instead of the semi-analytical approach used in this work. Focusing on very small clusters composed of only a few nodes with shared-memory capability, Eichner et al. (2009) demonstrated how to exploit multicore processor efficiently in simulations of morphologically detailed neurons, while an analysis concluded that real-time simulations of medium sizes plastic networks are not feasible on small clusters of CPUs, but will require accelerators or dedicated hardware (Zenke and Gerstner 2014). The work on the multisplit method (Hines et al. 2008) demonstrated that efficient acceleration of individual neurons on single compute nodes is difficult beyond a restricted number of cores, and recent work on micro-parallelism (Magalhaes et al. 2019a; 2019b) has found significant limitations to strong-scaling due to Amdahl’s law.

Several GPU implementations of brain tissue simulations have been proposed in the literature (Brette and Goodman 2012; Fidjeland et al. 2009; Yavuz et al. 2016; Kumbhar et al. 2019b). A comparison between GPUs, HPC hardware and neuromorphic hardware found that, under certain conditions, GPUs can beat neuromorphic hardware in terms of energy efficiency but not an HPC server in terms of performance (Knight and Nowotny 2018). However, the authors did not perform a detailed performance analysis nor used a performance model to explain this comparison, thus providing valuable yet anecdotal evidence. Their work is tightly linked to the neuron model, the configuration of the simulation as well as the hardware being analyzed.

In the process of creating the neuromorphic hardware SpiNNaker, the designers were deeply interested by the consequences of their decisions in terms of performance. An analysis showed that, in the context of real-time simulations, SpiNNaker is inherently limited in its scope to a restricted subset of synaptic formalism, because the single node’s memory bandwidth puts a hard limit on the number of synaptic parameters that can be streamed at every timestep (Painkras et al. 2013). In designing the network for SpiNNaker, a performance model was used to determine the ideal topology and network connectivity (Navaridas et al. 2012), proving that performance modeling can be an extremely valuable tool in optimization and co-design for brain tissue simulations.

## Materials and Methods

**Fig. 1:**
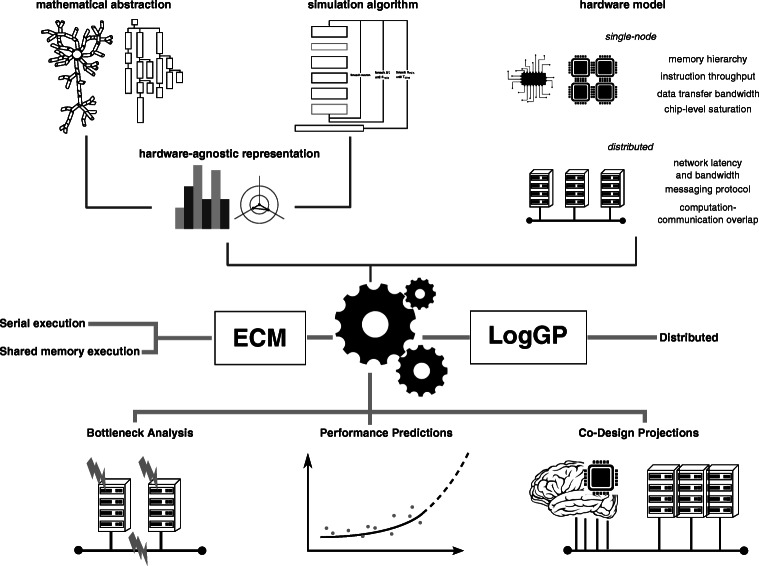
Comprehensive performance modeling of brain tissue simulations. An hardware-agnostic representation of the *in silico* model is obtained by combining detailed information about the mathematical abstraction, such as e.g. the representation of neurons and its implementation as data structures, or the formulation of the differential equations at the basis of the temporal and spatial dynamics, as well as the simulation algorithm and the dependencies between different simulation phases. This is combined with an abstract representation of the hardware based on a few key parameters, as well as a detailed understanding of the software implementation and the execution of the flow of instructions on the reference hardware, to obtain runtime predictions based on the ECM model for serial and shared-memory execution, and on the LogGP model for interprocess communication. Once our performance model is validated, we use it to predict the performance of brain tissue simulations in multiple configurations, analyze bottlenecks through introspection of the model and provide informed guidelines for the co-design of future hardware

We develop our analysis of the performance landscape in a two-step process as show in Fig. 1: first we identify relevant *in silico* models and experiments from the literature that constitute a representative sample of state-of-the-art models and algorithms, and present a set of hardware-agnostic descriptive metrics that can give a first insight on their performance properties; then we intersect the hardware-agnostic description with a model of the hardware platform, by extending and adapting well-established performance models to neuroscientific simulations use cases.

### In Silico Models and Experiments

**Fig. 2:**
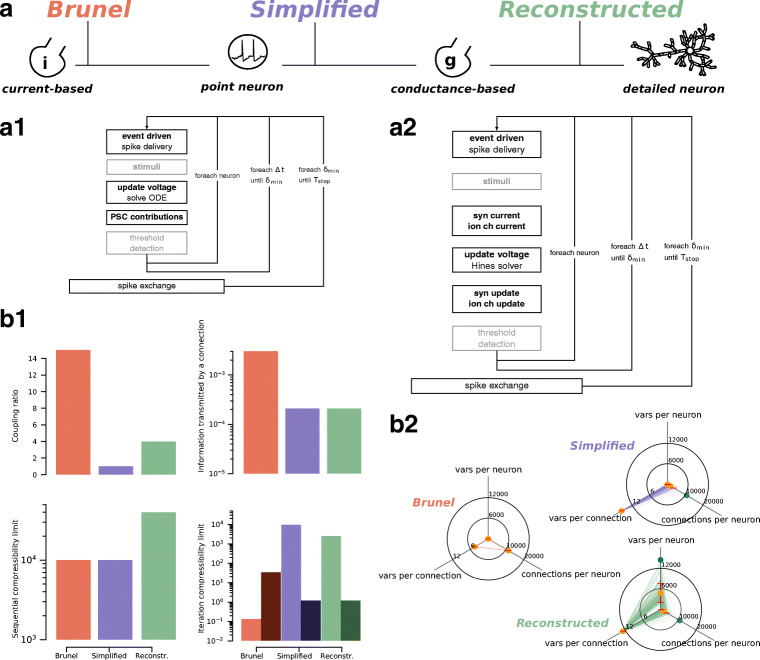
In silico models and experiments. Presentation and summary of the *in silico* models and experiments examined in this paper. **a** Color-coding for the three *in silico* models and salient features: in red the I-based point neuron *Brunel* model, in purple the G-based point neuron *Simplified* model and in green the G-based detailed neuron *Reconstructed* model. **a1,a2** I-based (resp. G-based) simulation algorithm. The simulation kernels within light grey boxes are included for completeness but are not considered in our analysis because they are not part of the computation loop. The larger boxes denote a synchronization point for distributed simulations. **b1** Hardware-agnostic metrics. Coupling ratio denotes the number of simulation timesteps before a global synchronization point. Information transmitted by a connection denotes the average number of variables transmitted via a connection during one minimum delay period. Sequential compressibility limit denotes the number of time iterations required to simulate one second of biological time. Iteration compressibility limit denotes the number of degrees of freedom updated in a $\delta _{\min \limits }$ interval. Lighter bars represent clock-driven updates, darker bars represent (average) event-driven updates. **b2** Breakdown of the unit size metric. This metric captures the memory footprint of *in silico* models, broken down in three components: number of variables to represent a single neuron excluding synaptic connections, number of variables to represent a connection, and number of connections per neuron. Orange dots represent mean values, red bars represent standard deviation and green dots represent maximal values. The lines represent actual samples from the model

The approach of identifying and singling-out recurrent computational patterns within a scientific field has been applied with great success in the domain of parallel computing, leading to the definition of the dwarfs of computing (Asanovic et al. 2009). In computational neuroscience, the review by Brette et al. (2007) proposed a similar approach and introduced some fundamental concepts, such as conductance based (G-based) and current based (I-based) formalisms for synaptic models, or point and detailed representations of neuronal morphology. Using the nomenclature introduced in that review, we base our analysis of the performance landscape on three published models, chosen as representative of the extent of neuron models covered in literature. We denote the set of these representative use cases as *in silico models and experiments*, and summarize their salient properties in Fig. 2a.

The *Brunel* model is a randomly connected network reproducing the property of balanced excitation and inhibition that can be observed in the brain cortex. It is based on integrate-and-fire (IAF) point neurons with I-based synaptic dynamics (Gerstner et al. 2014; Brunel 2000). As a representative example of this model, we consider here a very large-scale implementation that served as a proof of concept for the feasibility of human brain scale simulations (Kunkel et al. 2014).

The *Reconstructed* microcircuit is based on the reconstruction of neocortical microcircuit from a mix of experimental data and first principles (Markram et al. 2015). This model uses morphologically detailed neurons and short-term plastic G-based synapses to capture a large amount of biological detail.

The *Simplified* model was obtained by reducing the detailed models of the Reconstructed microcircuit to pointwise generalized integrate-and-fire (GIF) models with similar transfer functions, while retaining the complexity from G-based synapses (Rössert et al. 2016).

The scope of this paper is restricted to the inference phase of simulating a neural network, thus neglecting the simulation of learning, because of the additional layer of complexity that would arise from including long-term plasticity in our analysis. Moreover, to allow for reproducibility and ease of modeling, we have switched the random synaptic release mechanism in the Reconstructed and Simplified models with a deterministic implementation of the same rule, based on an average representation. This allows us to remove the uncertainty in the performance model due to probabilistic release, as well as the overhead from the random number generation, ultimately leading to better accuracy in the performance model. Although synaptic noise can be considered a primary driver of cortical dynamics (Nolte et al. 2019), we consider the complexity associated with random number generation in performance modeling outside of the scope of this paper.

#### Simulation Algorithm

We identify similarities and differences between the simulation algorithm of the *in silico* models and experiments. The first important remark is that the phases that make up the complete algorithm depend only on the type of synaptic formalism, and not on the level of morphological detail in the neuronal abstraction. Thus in Fig. 2a we present two algorithm skeletons: one for I-based models and one for G-based models. Moreover, regardless of the *in silico* model, there are always three nested loops: the outer one determines when global synchronization and communication of spike events happens; the central loop iterates over the simulation timesteps between two global synchronization events; the inner loop updates the state of each neuron by one timestep. In the I-based simulation workflow one needs, in addition to the event-driven integration of synaptic events, only to update the voltage of individual neurons by solving a few simple ordinary differential equations (ODE) and to update for each neuron the total post-synaptic current (PSC) contributions. In the G-based model, a linear system of equations must be solved for each neuron and each timestep to updated the voltage. In addition, for every synapse and ion channel one must compute its corresponding contribution to the matrix in the so called *current* kernels as well as update its state in the state kernels.

#### Hardware-Agnostic Metrics to Describe *in silico* Models and Experiments

As a first approach to navigating the performance properties landscape of *in silico* models and experiments we propose a collection of hardware-agnostic metrics that can be computed directly from inspection of the model specifications. These metrics have the same value regardless of the underlying simulation hardware, an interesting property if one wants to compare *intrinsic* model features. To set a common ground on which we define these metrics we identify the following features shared by all *in silico* models and experiments: i) all neural networks can be represented by a graph where neurons are nodes and synaptic connections are edges; ii) synaptic connections can be approximated by a perfect delayed transmission of information; iii) simulation algorithms include a clock-driven portion to integrate neuronal states and an event-driven portion to integrate synaptic events; iv) all simulation algorithms considered here follow the Bulk Synchronous Parallel (BSP) paradigm, where computation phases carried out independently by each parallel rank alternate with global synchronization steps, happening at fixed time intervals called minimum network delay (denoted by *δ*_*min*_). We are thus excluding from this analysis asynchronous communication schemes (Ananthanarayanan and Modha 2007; Magalhães and Schürmann 2019), variable timestep schemes (Lytton and Hines 2005) and models that explicitly represent axons (Kozloski and Wagner 2011).

We evaluate the performance metrics of *in silico* models on three aspects: memory, serial complexity and information propagation. In the memory dimension we consider aspects of the model that can affect the memory capacity footprint such as the number of parameters and degrees of freedom required to represent a neuron. Specifically we count the number of state variables and parametes per neuron, the number of state variables and parameters per connection and the fan in (i.e. the number of incoming connections) per neuron. In the serial complexity dimension we consider aspects tied to sequential iterations such as timestep and the number of state variables updated at each iteration. We define the *sequential compressibility limit* as the inverse of the timestep Δ*t* and the *iteration compressibility limit* as the number of state variables updated in a single time iteration. Finally, in the information propagation dimension, we consider aspects tied to communication of information between neurons such as the frequency at which a global synchronization must happen and the amount of information exchanged in this step. Here we define the *coupling ratio* as the number of timesteps that can be taken before a global synchronization point must happen, given by the formula $\frac {\delta _{min}}{\Delta t}$, and the *information transmitted by a connection* as the number of variables communicated by a connection on average during a minimum network delay period.

Figure 2b reports the values of the hardware-agnostic performance metrics obtained by hand-counting the relevant quantities in the published *in silico* models. Each of the metrics described above can be associated to one or more performance aspects and hardware features. For example, the low values of the coupling ratio for the Simplified and Reconstructed model can be associated to poor strong scaling properties, while the large information transmitted by a connection for the Brunel model translates to higher pressure on the network interconnectivity hardware. Concerning time iterations, the large event-driven component of the iteration compressibility limit for the Brunel model points to the fact that it could potentially be bounded by hardware latency aspects (either memory latency or critical paths in the execution) as well as dynamic imbalance, while the Simplified and Reconstructed model are more likely affected by throughput of hardware features. The large number of connections per neuron in the Brunel model and the large number of variables to represent a neuron in the Reconstructed entail that these *in silico* models will be bounded by memory capacity. Finally, the large variability of individual neurons in the Reconstructed model poses a potential static load-balancing problem, as was empirically found in Kumbhar et al. (2018) in the context of manycore processors.

### Analytical Performance Modeling of Brain Tissue Simulations

The metrics described previously provide an insightful summary of the performance profile of *in silico* models and experiments, but lack the power to give quantitative performance predictions and the connection with specific hardware properties. Therefore, we use performance modeling as a way of bridging the gap between biophysical models, simulation algorithms and hardware specifications. In particular, we split the performance prediction in a single-node component and an interprocess communication component. We address the single-node performance modeling using the Execution-Cache-Memory (ECM) model (Treibig and Hager 2010) and the interprocess communication part using the LogGP model (Alexandrov et al. 1997). Both are well-established approaches that have been extensively validated on several hardware platforms (Hager et al. 2016, 2018; Hoefler et al., 2009), however significant work is required to extend and adapt them to the simulation kernels in our analysis, for example accounting for indirect memory accesses in the single-node predictions and the representation of spikes in the communication component. Details for the extension and validation of the ECM and LogGP performance models are provided in the Supplementary Material S1.1 and S1.2.

#### Single-Node Performance Model

The ECM model uses a *grey-box* mixed approach combining an analytic formulation with some phenomenological input, and outputs a runtime prediction at the granularity of individual clock cycles (Treibig and Hager 2010). Since its introduction it has been refined and validated on modern Intel and AMD multicore architectures (Hofmann et al. 2017, 2018; Stengel et al. 2015) . To compute the ECM performance model for serial execution one must first define several contributions to the runtime of a given loop, such as: the in-core execution time assuming data is already loaded in registers *T*_*OL*_, the time needed to load data into registers from the L1 cache *T*_*nOL*_, the data traffic time between caches *T*_*L*1*L*2_,*T*_*L*2*L*3_ and the data traffic time from main memory *T*_*L*3*Mem*_. Data traffic times are usually computed combining an estimation of the data traffic with the bandwidth of the relevant data link. *T*_*OL*_ and *T*_*nOL*_, on the other hand, can be computed by hand but are typically extracted using code analysis tools such as Intel’s IACA (Intel 2017). These contributions must be combined to obtain two quantities: *T*_*core*_ and *T*_*data*_, representing the time that the loop would spend in core execution if data were instantaneously available, and the time required to move the data across the memory hierarchy, respectively. One of the core assumptions of the ECM model is that these two quantities can overlap, therefore single-thread runtime predictions can be obtained using the formula

1 $$ T = \mathop{max}\left( T_{core}, T_{data}\right). $$

The ECM model is based on the full-throughput assumption, thus neglecting any latency effects in the execution. This assumption greatly simplifies the analysis by removing the need for an extremely detailed understanding of the execution flow while at the same time providing *insight through failure* for situations in which the program execution is the bottleneck. In this context, a particular kernel will be categorized as **core-bound** if *T*_*core*_ > *T*_*data*_, and **data-bound** otherwise. Note that these definitions apply to the serial execution. To obtain a performance prediction for parallel execution, the ECM model assumes that performance scales linearly with the number of threads, until a bottleneck from a shared serial resource is used, typically the memory interface (Hofmann et al. 2015). The ECM also provides a formula for computing the **saturation point**, i.e. the number of shared memory threads at which saturation of the memory bandwidth occurs for a given kernel.

We computed the individual ECM dimensions and the corresponding runtime predictions for all clock-driven kernels of the *in silico* models, and report them in Supplementary Table S4. Moreover, we conducted a thorough validation of our runtime predictions and report the results in Supplementary Table S5 and Supplementary Fig. S10.

#### Spike Delivery Kernel

The performance modeling of the spike delivery kernel presents several challenges that warrant the need for a separate treatment. In terms of algorithm design, all state-of-the-art software use some sort of priority queue or ring buffer to store synaptic events to be delivered within a timestep. For modeling and benchmarking, we separate the operations related to the bookkeeping of events inside the queue from the actual kernel execution, and we only consider the latter, because the scope of our analysis is restricted to computational and communication kernels.

When a spike is received, the postsynaptic process must integrate its effects in the state of the target neuron or synapse. In I-based synapses this amounts to increasing a spike counter by the relative weight of the connection, while for G-based synapses an equivalent quantal update of the synaptic states must be computed, a computationally expensive procedure since the *in silico* models considered in this work all contain a short-term plasticity model.

The spike delivery kernel is characterised by erratic memory accesses, because the order of activation of synapses is unpredictable. We always consider the worst possible case in which every spike to be delivered could not be cached and thus must come from main memory. We assume that a full cache line of data needs to be brought in from memory *for every data access*, since the unpredictable order of activation of synapses renders data prefetching and data blocking largely ineffective. We consider that only accesses to synapse-specific data are non-contiguous and thus require a full cache line (64 B) of data to be transferred for every memory request.

Estimating the runtime proves to be a very challenging task. We find that the naïve approach of multiplying the DRAM latency by the number of non-contiguous accesses yields very pessimistic predictions. This can be attributed to the fact that, since spikes are independent, it is not necessary to wait until one spike has been processed before issuing request for the data of the next spike. It thus seems that the spike delivery kernel’s performance is determined by the number of concurrent, independent data requests that can be handled by the processor and memory. This is different from the classical purely latency bound kernels in which the CPU is only allowed to begin a loop iteration after the previous one is fully completed. The number of independent memory requests that can be handled concurrently is known as memory level parallelism (MLP) and allows to mitigate the performance impact of memory latency by allowing multiple accesses in parallel (Levinthal 2014). For shared memory parallelism, we assume that performance scales linearly with the number of threads until the bottleneck of memory bandwidth is reached. Details about the runtime prediction and validation are provided in the Supplementary Material S1.1 and Supplementary Fig. S11.

#### Spike Exchange in Brain Tissue Simulations

All the *in silico* models and experiments considered in this paper are based on the Bulk Synchronous Parallel (BSP) model (Valiant 1990), which prescribes a clear distinction between an *on-node computation* phase (happening in a distributed parallel fashion) and an *inter-node communication* phase. For brain tissue simulations, the inter-node communication phase corresponds to the spike exchange step in Fig 2a. Moreover, we make the assumption that the distributed processing is implemented in MPI, because it represents the current state of practice in the HPC community. Throughout this work, we maintain the nomenclature of shared memory threads and distributed ranks. When we use the generic term of parallel processes, we make the assumption that shared memory parallelism capabilities are always exhausted before distributed memory parallelism. Details on our application of the LogGP model to the spike exchange algorithm, as well as on the reference hardware, are provided in the Supplementary Material S1.2.

In all the state-of-the-art simulators, the spike exchange step is implemented by a blocking collective call, typically a variant of the Allgather operation. This entails that all the parallel ranks have, at the end of the communication step, knowledge of all the spikes produced by the simulation during the last minimum network delay period. Recent work has shown that at extremely large scales, this implementation can become prohibitively expensive in terms of memory requirements, and proposed to use instead the Alltoall operation to deliver spikes only to the ranks where they are required (Jordan et al. 2018). Other alternative implementations have been suggested, using nonblocking point-to-point communication (Ananthanarayanan and Modha 2007) or spatial decomposition (Kozloski and Wagner 2011). All these fall outside of the scope of this paper, which is focused on small-to-medium cluster sizes and well established, widely used software solutions.

#### The LogGP Model for Interprocess Communication

We use the LogGP model (Alexandrov et al. 1997) to predict and explain the performance of the spike exchange simulation step. The LogGP model is an extension of the LogP model (Culler et al. 1993) that uses an additional parameter, the gap per byte denoted *G*, as a way to account for the sending and receiving of long messages. We refer the interested reader to the Supplementary Material S1.2 for more details. The main features of all models based on LogP is that their parameters are easily relatable to hardware characteristics, thus ensuring a high degree of interpretability. In the LogGP model, the cost of sending a single message of size *m* bytes is given by two contributions: a latency contribution corresponding to the time it takes for the first byte of the message to reach its destination, and a bandwidth contribution corresponding to the throughput at which messages can be communicated through the interprocess network. One of the main insights in the LogGP model is that, under certain circumstances, CPU-side operations such as copying of data can overlap with network-side operation such as data sending.

Collective communication operations are difficult to model because different algorithms can be used to disseminate the messages across the network, and the choice of which one to use can happen dynamically and transparently to the user, depending on several performance factors. A review mentions however that the ring algorithm is the most commonly used, especially for large messages, and thus we base our predictions on this paradigm (Thakur et al. 2005). In the ring algorithm, the number of times in which parallel processors establish a network connection and start exchanging data is proportional to the total number *P* of parallel ranks in the simulation, therefore the latency term of the total cost of the collective communication is expected to scale linearly in *P*. Conversely, in the recursive doubling algorithm this term scale logarithmically, while for both algorithms the total amount of communicated datais the same, and thus the bandwidt term for both algorithms is expected to be the same. Using this information, we adapt the formula proposed originally by Mamadou et al. (2006) for recursive doubling, to account instead for the ring algorithm, as explained in equation (S10) in the Supplementary Material. In our modeling and validation we always consider that the number of spikes communicated by each rank is roughly homogeneous. The validation of our interprocess communication runtime predictions are provided in the Supplementary Fig. S12.

### Hardware Models

Our performance modeling methodology requires a detailed abstraction of the hardware to obtain accurate runtime predictions. In this work we use the Intel(R) Xeon(R) Gold 6140 Skylake processor as reference for the single-node hardware, We provide in Supplementary Table S3 the values of the hardware characteristics that are most relevant for our performance modeling methodology. while for distributed communication we use as reference a vendor (HPE) provided MPI implementation, based on MPT 2.16 and the MPI 3.0 standard, over an Infiniband EDR 100 GB/s fabric. This architecture is highly representative of modern, state-of-the-art high performance computing clusters of CPUs. For validation purposes, we execute benchmark simulations on the reference hardware by inputting random synaptic inputs at a frequency of 1Hz into every cell within a network of disconnected neurons. All the benchmarks were executed multiple times under the same conditions (typically around 10 runs), and we define the error (or margin of error) as the ratio of the difference between the median measurement and predicted runtime divided by the median measurement. For validation and benchmarking we use the CoreNEURON implementation as reference (Kumbhar et al. 2019b).

Using our reference Skylake hardware as basis, we also develop the concept of strawman architectures: models of the hardware that do not necessarily reflect reality perfectly in every detail, but that capture the most salient hardware properties and can be used to explore the design space easily. We consider in this work another multicore CPU server based on the AMD Naples architecture, a manycore Intel Knight’s Landing (KNL) architecture and a GPU-like architecture inspired by the Nvidia Volta V100 GPU. For each strawman architecture, we took the amount of shared-memory parallelism, the memory bandwidth, the clock frequency and the cache hierarchy from nominal values or published studies (Hofmann et al. 2019; Jeffers et al. 2016; Jia et al. 2018). Other important features such as the instruction throughput and the memory level parallelism and latency, that require extensive benchmarking, could not be obtained directly. Instead, we opted to infer them from the corresponding values in the reference Skylake architecture, taking the memory level parallelism and latency as they were, and scaling the instruction throughput to the appropriate level of vectorization. For simplicity, we considered only the high-bandwidth memory on the KNL architecture, and we treated individual streaming multiprocessors in the GPU-like architecture as shared memory threads with SIMD registers of 32 double-precision floating point variables width, connected directly to the global GPU memory.

## Results

Our goal is to provide a quantitative appraisal of the performance landscape of brain tissue simulations and analyze in detail the relationship between an *in silico* experiment, the underlying neuron and connectivity model, the simulation algorithm and the hardware platform being used. We carry out this analysis with the tool of performance modeling, allowing us to quantify and explain performance bottlenecks without the need of time-consuming and narrowly-scoped benchmarks.

### Serial Performance Profile

We uncover the fundamental performance properties of simulations of biological neurons by examining the serial performance profile. We counted by hand the number of floating point operations (flop) and the data traffic required to simulate one neuron receiving synaptic events at a rate of 1Hz for one second of activity, for different modeling abstractions. Table 1 summarizes the results. As expected, the flop and data requirements grow with the amount of biological detail in the model, leading to a factor 10^4^ difference between the Brunel and Reconstructed model. Interestingly, the arithmetic intensity of all the models is roughly the same, in the order of 10^− 1^. This may be ascribed to the fact that the arithmetic intensity of the differential equations being solved in each model is essentially very similar, and only the number of equations to represent a neuron within each modeling abstraction is different. We also looked at the total amount of memory required to store a neuron’s states and parameters, and found that the Simplified model’s strategy of lumping synapses allows it to greatly optimize the memory capacity requirements. Finally, we conclude the analysis by using our model to predict the simulation performance, measured in biological seconds per elapsed wallclock second to simulate a single neuron receiving synaptic events at a rate of 1Hz. A factor 10^4^ difference in the performance of the Brunel and Reconstructed model mirrors perfectly the difference in flop and data traffic requirements observed above.

**Table 1:** Average number of flop and data traffic per neuron to advance its state by 1 second of simulated time

	Brunel	Simplified	Reconstructed
Flop	2.3 × 10^5^	1.7 × 10^7^	8.5 × 10^9^
Data Volume [MB]	1.0	1.3 × 10^2^	3.1 × 10^4^
Arith. Int. [Flop/B]	2.3 × 10^− 1^	1.3 × 10^− 1^	2.7 × 10^− 1^
Mem. Capacity [KB]	2.7 × 10^2^	10	2.9 × 10^3^
Perf. [sim s / wall s]	3.4 × 10^3^	1.7 × 10^2^	3.4 × 10^− 1^

Arithmetic Intensity is defined as the ratio of flop per data volume. We also report the predicted serial simulation Performance, measured in simulated seconds per elapsed wallclock second for a single neuron receiving synaptic events at a rate of 1Hz. The requirements in this Table are computed considering only the data structures strictly relevant to computation, thus neglecting overhead from implementation details such as MPI buffers, data structure representation, memory padding, etc

**Fig. 3:**
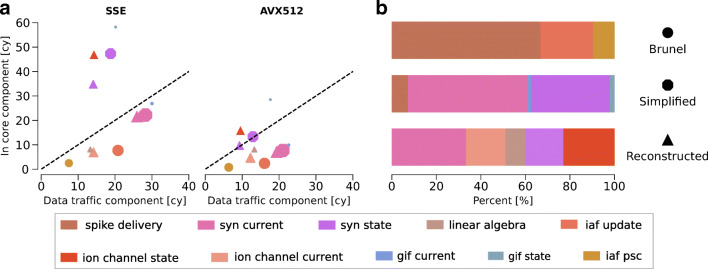
Predicted serial performance characteristics of clock-driven computational kernels in brain tissue simulations. We predict the serial runtime of *in silico* models as a sum of their individual kernels on the reference SKX AVX512 architecture. **a**: *T*_*core*_ and *T*_*data*_ components of the clock-driven kernels from brain tissue simulations. The dashed black line delineates the boundary between core-bound kernels (over the line) and data-bound kernels (under the line). Marker type denotes the *in silico* model whence the kernel is taken, while marker size is proportional to the relative importance of the kernel in the total runtime. **b**: breakdown of the relative importance of individual kernels over the total serial runtime

We complete our above analysis with a detailed characterization of the performance profile of simulation kernels based on the reference hardware. At first we consider the *T*_*core*_ and *T*_*data*_ components of all the individual kernels that constitute the simulation algorithm of different modeling abstractions. Figure 3a demonstrates that most kernels are data-bound on the reference architecture, even when in the case of short vector registers (SSE). When larger vector registers are used (i.e. AVX512), the *T*_*core*_ component can be greatly improved while the *T*_*data*_ remains roughly the same. This is an indication that the performance of core-bound kernels can be improved by vectorization, until the kernel becomes data-bound. In addition, we observe that when using the vectorization hardware to its fullest potential (i.e. AVX512) several kernels lie on the boundary between core-boundedness and data-boundedness. This represents a balanced profile where the ratio of data traffic and computation matches the design space of the hardware. To draw conclusions about the full models, we need to intersect this information with the relative importance of the individual kernels on the total runtime. The serial performance of G-based models is dominated by the state and current kernels, in roughly equal parts. Thus we conclude that G-based models are mainly data-bound. In the I-based model the most time consuming kernel is the event-driven spike delivery. This implies that, while the clock-driven portion of the I-based model is definitely data-bound, the serial performance hardware bottleneck of the whole neuron model is memory-level parallelism and latency.

### Memory Bandwidth Saturation in Shared-Memory Execution

One of the most common simulation configurations involves scaling the number of neurons until the memory capacity limit is reached. This configuration has been used as proof-of-concept for brain tissue simulations to the scale of brain regions and even the full brain and constitutes a fundamental tool for neuroscientists to simulate networks whose sizes are representative of the neural systems they are studying (Ananthanarayanan et al. 2009; Jordan et al. 2018; Izhikevich and Edelman 2008).

#### Memory Bandwidth Limits Shared-Memory Parallelism

**Fig. 4:**
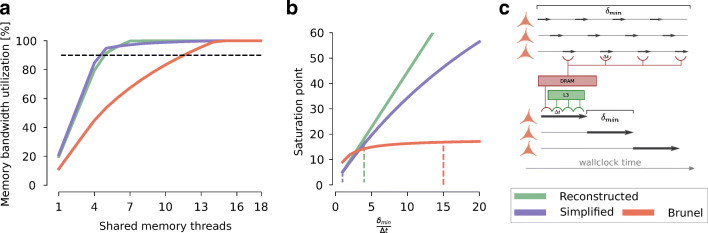
Predicted shared-memory performance characteristics. We predict the shared-memory runtime of *in silico* models as a sum of their individual kernels on the reference SKX AVX512 architecture. **a** Percentage of memory bandwidth utilization as a function of the number of shared memory threads. The dashed black line denotes the threshold of 90% utilization. **b** To mitigate the effect of memory bandwidth saturation, a smart ordering of time and neuron loops is implemented by state-of-the-art simulators, as shown in the diagram on the right. We plot the number of threads required to reach saturation of memory bandwidth as a function of the coupling ratio. Different coupling ratios were enforced by keeping the Δ*t* fixed to each model’s published value, and changing the $\delta _{\min \limits }$ accordingly. Dashed lines represent the actual published values for the coupling ratio. **c** schematic representation of the loop ordering optimization to improve cache reuse. The top shows the naïve implementation: each neuron, represented by an horizontal line, is advanced by a single timestep, as shown by the short black arrows. In this case, every time a neuron’s state is advanced by one timestep data must be fetched from the main memory (red lines), since the caches will be overwritten by the data from other neurons at the same timestep. The bottom shows the optimized version: each neuron is advanced by several timesteps (longer black arrows) until it reaches a $\delta _{\min \limits }$ boundary. In the optimized version data must be fetched from main memory only during the first timestep, while consequent operations can reuse the data for the same neuron immediately (green lines represent data coming from the L3 cache)

Modern architectures are typically designed with memory bandwidth as the most relevant bottleneck for shared-memory parallelism (McCalpin 1995). This means that if all the shared memory parallel threads are used, it is very likely that performance will be bounded by the memory bandwidth. Indeed, this has been demonstrated to be the case for simulations of detailed neurons (Cremonesi et al. 2019) and strongly suspected in the case of point neurons (Zenke and Gerstner 2014). To verify this hypothesis we compute the memory bandwidth utilization for the three *in silico* models considered in this work. The results are shown in Fig. 4a. We find that all models pass the threshold of 90% utilization well before all available parallel threads are utilized, meaning that memory bandwidth is indeed a bottleneck under the assumption that data must be pulled from main memory *at every time iteration*. However, we surprisingly also find that, regardless of the level of morphological detail, G-based models share a similar pattern of early saturation while the I-based IAF model requires slightly more parallelism to achieve memory bandwidth saturation. In G-based models this is explained by the dominance of synaptic and ion channel current kernels which determine the early saturation pattern, whereas in the I-based model the saturation is driven by the memory latency effect on the spike delivery kernel.

#### State-of-the-Art HPC Memory Chips Can Sustain Fast Simulations of the Brunel and Simplified Models at Full Saturation

From a practical point of view, in addition to analyzing the scaling behaviour of simulations, computational neuroscientists are also interested in predicting the actual runtime for a given model. Thus we predict that the simulation performance, under the assumption that *memory bandwidth is fully saturated*, is 1.6 × 10^4^,7.9 × 10^2^ and 1.7 simulated seconds per wallclock second per neuron, for the Brunel, Simplified and Reconstructed model respectively. Our results indicate that the modern, fast memory chips on the reference architecture are able to sustain faster-than-real time simulations of up to roughly 10^4^ neurons in the Brunel model, and 10^3^ neurons in the Simplified model, while faster-than-real time simulations of the Reconstructed model on the reference hardware are predicted to be theoretically possible only by a narrow margin, and in practice probably impossible. Performance predictions under the memory bandwidth saturation assumption represent a theoretical upper limit on the achievable performance through shared memory parallelism. The following paragraph explains how this limit can be overcome through algorithmic improvements.

#### Ordering of Loops to Avoid Memory Bandwidth Saturation

State-of-the-art simulators employ a specific ordering of the loops over neurons, timesteps (Δ*t*) and minimum network delay steps ($\delta _{\min \limits }$) to minimize the impact of memory bandwidth by maximizing data locality. This optimization is summarized in Fig. 4c. Throughout this work we make the conservative assumption that, when using the loop ordering optimization, data must be fetched from main memory on the first timestep and from the L3 cache on consecutive timesteps until a minimum delay barrier is reached. The number of timesteps within a minimum delay period has of course a great influence on the effectiveness of this strategy in terms of reducing pressure on the memory bandwidth. To quantify this, we compute the number of threads to reach saturation – *n*_*satur*_ – and plot the results in Fig. 4b as a function of the coupling ratio defined by $\frac {\delta _{\min \limits }}{\Delta t}$. In G-based models, there is an almost linear relationship between the coupling ratio and *n*_*satur*_, indicating that investigating ways to increase the coupling ratio could be highly beneficial for parallelism. Note that, in this regard, increasing the coupling ratio by decreasing Δ*t* presents a performance tradeoff: it allows more parallelism but increases the computational requirements (number of iterations) of the model. Conversely, while the minimum network delay is obviously a fixed parameter of the network that cannot be arbitrarily changed, methods that experimented with a per-neuron delay, instead of a network-wide minimum delay, demonstrated significant speedup (Magalhães and Schürmann 2019). The relationship between coupling ratio and *n*_*satur*_ for I-based models is bounded by a relatively small limit of roughly *n*_*satur*_ ≤ 17, above which no additional parallelization is predicted to provide any benefit. This is explained by the fact the spike delivery kernel, in virtue of its event-driven nature, is unaffected by the benefits of the coupling ratio. Since our assumption is that data for this kernel must always come from main memory, as soon as it becomes the dominating performance factor and it reaches saturation, it inhibits any benefit from parallelism.

### High Speed Single-Node Simulations

Another widespread simulation regime is focused on simulating a fixed size network as fast as possible. We call this the constant problem size regime, and within it we make the assumption that the optimized loop ordering is always implemented to minimize the pressure on the memory bandwdith. One use case falling within this performance regime is real time simulations, in which one second of simulated time requires at most one second of wallclock time. Currently, on the one hand it is unclear whether real time is realistically achievable on modern hardware (Zenke and Gerstner 2014), and on the other hand special hardware that breaks this limit by design has already been conceived and tested for small networks (Aamir et al. 2018).

#### Memory Bandwidth Dominates the Shared-Memory Strong Scaling of Brunel and Simplified Models, while a Mix of Hardware Features Influences the Performance of the Reconstructed Model

Simulations of networks comprising a small number of neurons can be advantageous because if the dataset can be fully contained in the CPU caches, superlinear speedup can be observed. Therefore we predict the simulation performance per neuron assuming the dataset could be fully contained in different levels of the memory hierarchy. For simplicity, we neglect the fact that some of these model and cache combinations are infeasible in practice, e.g., due to the memory footprint of a single neuron in the Reconstructed model exceeding the L1 cache size. The performance predictions are reported in Table 2 assuming all available threads in the reference architecture (18 in total) are being utilized. Note that the reported performances are better than the theoretical limit computed in the previous section by assuming memory bandwidth saturation. While this may seem counterintuitive, it can be readily explained by the use of the loop ordering optimization, which allows to perform multiple time iterations without the need to pull data from DRAM.

**Table 2:** Predicted performance per neuron without the memory bandwidth saturation assumption, considering all available threads are used

	Brunel	Simplified	Reconstructed
performance (DRAM)	5.2 × 10^4^	7.9 × 10^2^	3.9
speedup in L3	2.5	4.7	1.8
speedup in L2	8.7	6.5	2.4
speedup in L1	33.9	6.6	2.4

Performance is measured in simulated seconds per wallclock second

**Fig. 5:**
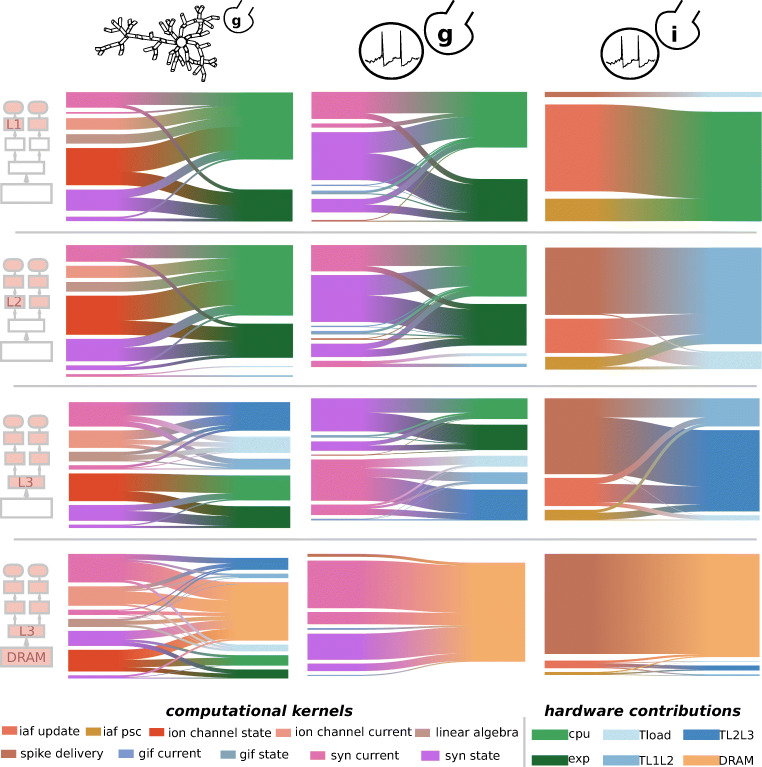
Predicted shared-memory runtime contributions from computational kernels and hardware features. We assume a single node of the SKX AVX512 and using the maximum number of threads (18 threads). We do not make the assumption of memory bandwidth saturation, but we assume that the loop ordering optimization is used. For each level of the cache hierarchy, we show the breakdown of the total runtime into computational kernels on the left of each box. Furthermore, we show the breakdown of the runtime, as well as the breakdown of individual computational kernels, into hardware contributions on the right of each box. Hardware contributions labels have the following meaning: CPU stands for the execution of non-memory access instructions in the core (excluding the exponential function), exp for the computation of exponential function, *T*_load_ for the execution of memory access instructions in the core, and the rest for the data traffic time of the relevant datapath

Figure 5 shows the predicted performance breakdown into simulation kernels as well as hardware features for all *in silico* models, assuming that the dataset fits in different levels of the memory hierarchy. When data is in the highest level of the cache hierarchy (L1), the most important kernels for all models are state update kernels, and the most relevant hardware feature is the CPU throughput. Additionally, in the G-based models the computation of the exponential (for updating the synaptic states) constitutes a significant portion of the overall execution time. As the dataset increases in size and is only able to fit in lower levels of the cache (L2 or L3) the predicted performance of the G-based models remains quite stable while that of the Brunel model degrades rapidly, although admittedly our model for the spike delivery kernel in caches might be highly optimistic. In practice, this could be an indication that the Brunel model is bounded by the data path while the G-based models are bounded by the maximum achievable flop rate. Our breakdown analysis confirms this, although for the reference architecture G-based models are best represented by a mix of core-bound and data-bound kernels, especially when the dataset fits only in the L3 cache. Complementarily, in G-based models the relative importance of the core-bound state update kernels gradually loses weight in favour of data-bound current kernels, while in the Brunel model the weight of the spike delivery kernel gradually increases, eventually becoming the most relevant kernel in the execution, as data moves further away from the CPU. In spite of this technique, both point neuron models are clearly dominated by the saturation of the memory bandwidth. In particular, the fact that memory bandwidth is the only factor in determining the performance of the Simplified model can be directly related to the fact that its coupling ratio has a value of 1, as shown in Fig. 2. The performance profile of the Reconstructed model is more diverse, and while 60% of the execution time is still dominated by memory bandwidth, the data transfers between the caches, arithmetic instructions, and throughput of exponential function evaluations also take up a significant portion of the runtime.

### Distributed Simulations

An effective strategy for improving simulation performance or to handle larger networks is to dedicate more hardware to the task, distributing the simulated neural network across multiple compute nodes. Here we consider two scenarios, based on the terminology introduced by Singh et al. (1993): a memory constrained scenario and a constant problem size scenario. In the memory constrained scenario the number of neurons is scaled proportionally to the available parallelism (i.e. the number of distributed ranks), while in the constant problem size scenario the number of neurons to be simulated is kept fixed. These scenarios translate respectively to the concepts of weak and strong scaling in high performance computing.

#### Performance Predictions of Distributed Brain Tissue Simulations

**Fig. 6:**
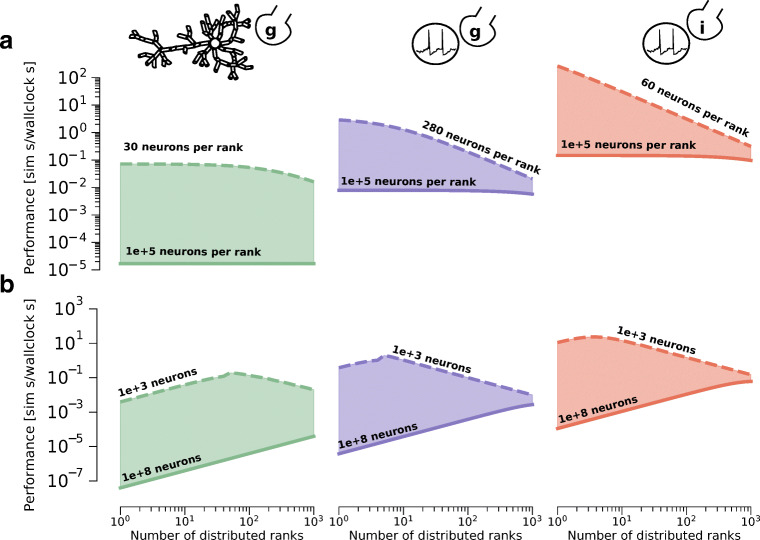
Performance of distributed scaling and most relevant hardware bottlenecks. The SKX AVX512 architecture with HPE Infiniband EDR is used as reference. **a** Predicted performance of the three *in silico models* in a memory constrained scenario. We consider different numbers of neurons per distributed ranks. The solid lines represent simulations with 10^5^ neurons per rank, while the dashed lines represent the estimated minimum number of neurons that is still larger than an L3 cache. The unit of performance is simulated seconds per wallclock second to simulate the whole network. **b** Predicted performance of the three *in silico models* in a constant problem size scenario. We consider different total network sizes. The dashed and solid lines represent simulations with networks of 10^3^ and 10^8^ neurons respectively. The unit of performance is simulated seconds per wallclock second to simulate the whole network

We predict the performance of *in silico* models in both scaling scenarios using our performance model, and present the results in Fig. 6. In the memory constrained scenario the simulation performance remains constant regardless of the total number of neurons as long as the number of neurons per rank is sufficiently large. In addition, as expected, the Brunel model has the best predicted performance, beating by up to a factor 10 the performance of the Simplified model and up to a factor 10^4^ the performance of the Reconstructed model. For small values of the number of neurons per rank, the performance of the Brunel model degrades with the amount of parallelism, while that of the Reconstructed model is roughly constant, exhibiting only a small degradation at large cluster sizes. In the constant problem size scenario, for all *in silico* models, as long as the network size is sufficiently large, the performance initially improves as we distribute the problem over increasingly more ranks. However, for all *in silico* models there exists a threshold number of ranks after which the benefits from adding hardware become less prominent. Interestingly the striking differences in performance between *in silico* models at small cluster sizes can be evened out quite significantly at large cluster sizes. For example, simulating a large Brunel network on 10 distributed ranks can be roughly four orders of magnitude faster than a Reconstructed network on the same hardware, but the difference between models goes down to two orders of magnitude at large cluster sizes. Scaling to larger cluster sizes after this threshold can be counter-productive, and even result in performance degradation.

#### Network Latency and Memory Bandwidth are the Main Bottlenecks in Strong Scaling

**Fig. 7:**
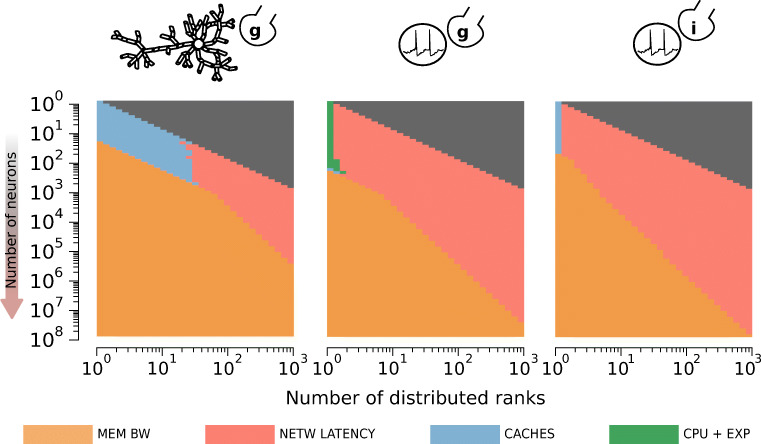
The SKX AVX512 architecture with HPE Infiniband EDR is used as reference. Most prominent hardware bottlenecks as a function of the total number of neurons (inverted *y* axis) and the number of distributed ranks (*x* axis) in the simulation. The grey areas denote a configuration that would require splitting of individual neurons, and are thus deemed outside the scope of this investigation.

Through introspection of the performance model, we are able to provide an explanation for the observed performance patterns. For each *in silico* model, we investigate the reasons for performance degradation by plotting the most significant hardware bottlenecks for all combinations of network size and cluster size in Fig. 7. We assume that the loop ordering optimization is being used. Even though we do not make the explicit assumption of memory bandwidth saturation, this hardware feature is still among the most relevant for all *in silico* models. Moreover, network bandwidth is never the dominating bottleneck for all *in silico* models and all configurations, while network latency always becomes the most important bottleneck at large cluster sizes. When network latency is the bottleneck we observe a corresponding degradation in performance as more parallel ranks are utilized. This can be directly attributed to the fact that network latency introduces a performance overhead that increases linearly with the number of distributed ranks. Thus we conclude that large-scale simulations are dominated by the latency of the collective communication, and that investigating spike communication strategies such as neighbourhood collectives (Jordan et al. 2018), non-blocking point-to-point schemes (Ananthanarayanan and Modha 2007), asynchronous execution (Magalhaes et al. 2019b) or custom hardware (Navaridas et al. 2012) will be essential to reach brain-scale simulations.

### Strawman Architectures and Hardware Design Decisions

A useful feature of our performance model is that it can be generalized to other architectures in a strawman fashion. In this section we focus on providing an educated guess on the performance profile of brain tissue simulations on hardware architectures made with fundamentally different design choices. For this goal we develop the concept of strawman architectures, i.e. models of the hardware that do not necessarily reflect reality perfectly in every detail, but that capture the most salient hardware properties and can be used to explore the design space easily.

**Fig. 8:**
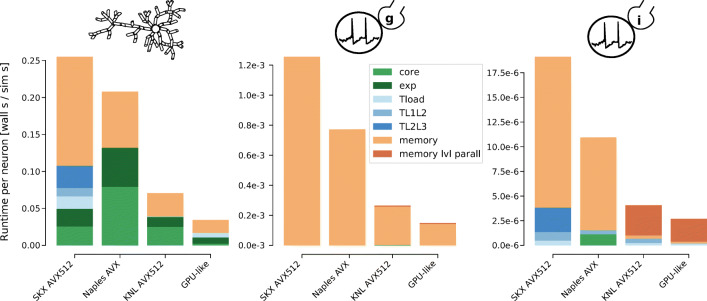
Breakdown of contributions to total runtime from individual hardware features. Bars represent the total predicted runtime on different strawman hardware architectures for the Reconstructed, Simplified and Brunel model on the left, center and right respectively. Each strawman architecture represents a simplified version of the target hardware, capturing salient hardware properties such as the amount of available shared-memory parallelism, the memory bandwidth, the clock frequency and the memory hierarchy. The rest of the hardware details, most notably the throughput of instructions in the core and the memory level parallelism and latency, were obtained by adapting the corresponding known values from the reference Skylake architecture. The meaning of individual hardware contributions is based on the corresponding ECM model dimension as in Fig. 5

Our strawman analysis predicts a speedup of 3x-5x when using the KNL manycore architecture and a speedup of 7x-9x when using the GPU-like architecture compared to the runtime on the reference SKX AVX512 architecture, for all models (Fig. 8). These results have been qualitatively confirmed in cross-platform performance studies (Knight and Nowotny 2018; Kumbhar et al. 2019a; Akar et al. 2019). In this work, however, we are able to dig deeper and identify substantial differences in the hardware bottleneck profiles of individual *in silico* models. The Reconstructed model, for example, is bounded by a mix of scalable features (e.g. arithmetic operations and exponentials) and non-scalable features such as the memory bandwidth. In particular, in architectures with a low clock frequency (KNL) or smaller vector registers (AMD Naples) the non-scalable components constitute more than half of the predicted execution time, indicating that more parallelism could potentially still be beneficial. On the other hand, the Simplified model is predicted to be bounded by memory bandwidth on all architectures. While improving the performance of such a model may seem a difficult task, our previous analysis has shown that improving the coupling ratio of the Simplified model would prove quite beneficial. Finally, The Brunel model is bounded mainly by memory bandwidth on the multicore server-like architectures, but appears bounded by memory level parallelism and latency on the manycore KNL and the GPU architecture. While a more detailed analysis based on actual memory latency values from each architecture would be required to confirm this, it points to an interesting tradeoff between the memory bandwidth and the available parallelism, and indicates that the Brunel model could potentially benefit from high amounts of shared-memory parallelism even with a relatively slower memory bandwidth.

### Effect of Model Parameters

**Fig. 9:**
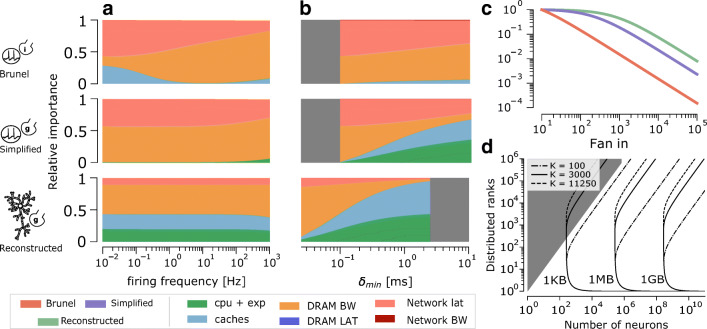
**a** Stacked plot of the mean relative contributions from hardware features as a function of the average firing frequency of neurons in the simulation. The mean was extracted by simulating 1000 randomly generated simulation configurations, defined by number of neurons and number of distributed ranks. **b** Stacked plot of the mean relative contributions from hardware features as a function of the $\delta _{\min \limits }$. The range of acceptable values for $\delta _{\min \limits }$ changes across different *in silico* models because they were computed as multiples of the model’s timestep, hence the greyed-out areas. **c** Number of neurons able to fit in 1 GB of memory, normalized by the memory requirements of a model with 10 incoming synapses, as a function of the average fan in per neuron. **d** Contour plot of predicted memory requirements of the connections table, as a function of the total number of neurons (*x* axis) and the number of distributed ranks (*y* axis). The contour levels corresponding to 1 KB, 1 MB and 1 GB are shown for different values of the fan in

Parameters of the *in silico* models have an important, yet often difficult to explain, impact on performance. Firing frequency is commonly cited as one of the most impactful parameters on simulation performance (Yavuz et al. 2016). Firing frequency affects communication by changing the size of the spike message as well as computation by changing the amount of events that must be integrated by neurons. By predicting the performance profile for different values of the firing frequency our analysis shows that, in the median case, the performance of the Reconstructed model is largely unaffected by this parameter, while in the case of the Simplified and Brunel model performance scales linearly with the firing frequency in simulations with large amounts of synaptic activity, as shown in the Supplementary Fig. S13. We further investigate how firing frequency affects the relative importance of hardware bottlenecks, and show in Fig. 9a that while the Reconstructed and Simplified model are largely unaffected by this parameter, the Brunel model’s performance profile becomes highly skewed towards the memory bandwidth at large values of the firing frequency. Interestingly, our analysis also shows that the minimum network delay also has a significant impact on performance, despite the fact that it does not change the total number of operations to be performed. In particular, for point neuron models there is a transition from a regime dominated by communication to one dominated by computation, while the Reconstructed model is dominated by computation for all values of the minimum network delay, as shown in Fig. 9b. Finally, we show how the average number of incoming synaptic connections presents a subtle tradeoff of increasing the computational load (thus decreasing performance) while at the same time increasing memory requirements, and thus potentially the amount of distributed parallelism required to simulate a given number of neurons (thus increasing performance). We examine two ways in which fan influences memory requirements: Fig. 9c shows the fraction of neurons that can fit in 1 GB of memory for different values of the fan in, solely by virtue of the additional parameters and state variables required to represent the corresponding synapses; Fig. 9d further investigates the memory requirements of the connection table, required by each distributed rank to determine whether a specific source neuron has any local postynaptic connections on that rank. We refer the interested reader to the Supplementary Material S2 for additional details in the analysis.

#### Synaptic Plasticity

The scope of this work is limited to the investigation of the performance properties of the inference phase of biological neural networks, assuming that the learning process, if any, is carried out in a separate experiment. However, using insight from our analysis, we can still show that synaptic plasticity would not likely change the performance profiles that we have identified in a dramatic way. First of all, given the event-driven and unpredictable nature of synaptic activation, it should be noted that plasticity kernels share the same memory access pattern as the spike delivery kernels, and are thus also potentially affected by memory level parallelism and latency. While long-term plasticity rules typically also involve computationally heavy operations, it is reasonable to assume that the memory level parallelism will be the dominant hardware bottleneck, given that the G-based spike delivery kernel has a similar profile. Therefore, we expect that in the Reconstructed and Simplified models the impact of synaptic plasticity rules would be negligible, since the event-driven portion of the runtime was already found to be small, and although synaptic plasticity would definitely increase it, it is unlikely that this would ultimately amount to a significant effect. In the Brunel model, which is dominated by event-driven computations, synaptic plasticity would simply exacerbate this profile as it shares the same bottleneck profile as the spike delivery kernel.

## Discussion

In this work we have delivered a quantitative characterization of the performance properties of different published *in silico* models at the core of state-of-the-art brain tissue simulations. Using a grey-box model that combines biological and algorithmic properties with hardware specifications we have identified performance bottlenecks under different simulation regimes, corresponding to a variety of prototypical scientific questions that can be answered by simulations of biological neural networks.

### General Purpose Computing has Sustained a Diverse Performance Landscape up to Now

Our results show that there exists a large diversity of performance profiles and bottlenecks that shape the landscape of brain tissue simulations, corresponding to the diversity of sizes and scales at which research questions in simulation neuroscience can be asked. Thus, our research highlights that the computational neuroscience community is currently greatly benefitting from the adaptability of general purpose computing, exploiting the ease of development and high performance capability to explore different areas of the modeling landscape.

### Memory Bandwidth and Network Latency Severely Limit Maximum Filling and Real Time Strong Scaling

Using a state-of-the-art HPC server CPU and cluster as a reference, our analysis revealed that all the *in silico* models saturate the memory bandwidth using quite a small number of shared memory threads. Even when algorithmic improvements are put into place to mitigate this effect we have identified that the coupling ratio, a dimensionless number that counts the number of timesteps in a mininum network delay period, strongly regulates the saturation of memory bandwidth and, in the extreme case of the Simplified model analyzed here, effectively prevents any benefit to be gained from the effort of developing a more efficient algorithm. Additionally, we discovered that it is not the level of morphological detail, but rather the formalism used to represent synapses, that is the most important factor in explaining the memory bandwidth saturation profile, with G-based models saturating much faster than the I-based model. Our analysis of strawman architectures has shown that the extremely fast memory bandwidth of current manycore architectures is currently able to sustain large amounts of shared-memory parallelism for all models, with performance improvements proportional to bandwidth improvements. In distributed simulations we identified the network latency, and not the network bandwidth, as the major bottleneck for scaling to very large networks or very large cluster sizes. This provides a new motivation and justification for the extensive efforts described in Navaridas et al. (2012) in designing a specific communication infrastructure for the SpiNNaker neuromorphic system.

### Model-Specific Features have a Significant Impact on Shared-Memory Performance

Inspection of our performance model allowed us to pinpoint which kernels, hardware specifications and model parameters have the largest impact on performance. The Brunel model based on the I-based formalism and IAF neurons is mainly bounded by the spike delivery kernel, which exhibits a good shared-memory scaling behaviour and, in the case of extreme strong scaling, a strong dependence on the inter-cache data paths for good performance. The two G-based models we analyzed, i.e. Simplified and Reconstructed, have a similar shared-memory scaling behaviour, mainly driven by the *current* kernels required to compute the contributions of individual synapses (and ion channels) to the membrane potential equation. However, while the Simplified model is 100% dominated by memory bandwidth, the morphologically detailed Reconstructed model is dominated partially (around 40%) by other hardware components such as caches and CPU throughput. Using strawman architectures, we have confirmed that these profiles are valid for a wide range of hardware design choices, with differences arising only when memory bandwidth is not fully saturated. It becomes clear that a performance model and a detailed performance analysis are fundamental tools to disentagle the complex web of relationships between *in silico* models, their software implementation and hardware concretization.

### Static and Dynamic Model Parameters Affect Performance in Significant but Subtle Ways

Finally, we examined the impact of model parameters on the performance profiles described above. We found that firing frequency, but surprisingly also minimum network delay, can have a large impact on determining which hardware features may constitute a performance bottleneck. For firing frequency it is obvious that larger values correspond to more operations required by the simulation algorithm, and thus a lower performance, but our analysis shows that different values of the firing frequency also change the relative importance of hardware features. Interestingly we found that the minimum network delay, in spite of it not affecting the total number of operations per simulated second, can have an effect on performance simply by shifting the importance of the hardware bottlenecks. We also found that the average number of incoming connections per neuron plays a subtle role in influencing performance. Trivially, a larger fan in increases the computational requirements of a single neuron. However, it also increases the memory capacity requirements, thus requring a larger degree of parallelism to handle the same network size. This creates a tradeoff between performance degradation arising from larger computational requirements and performance improvement from parallelism requirements.

### Limits and Future Improvements

In this work we have concentrated solely on the aspect of maximizing performance, without considering limitations such as cost or energy. However, it must be stated that energy efficiency is a central issue in the computational neuroscience community, and one of the main selling points of neuromorphic hardware (Cassidy et al. 2014; Stromatias et al. 2013). Therefore, a meaningful extension to this work would be to incorporate a model for power consumption alongside performance prediction, as a way to constrain the feasibility and efficiency of certain simulation configurations. To achieve this, one could exploit already established power consumption models that are easily integrated with the ECM and have been shown to provide valuable insight into the power and performance properties of simulation kernels (Hager et al. 2016; Hofmann et al. 2018). Moreover, our analysis has been focused on computational and communication kernels, effectively neglecting other aspects of simulation performance such as generation of stimuli, random number generation and managing the queue of synaptic events. While these kernels constitute necessary steps in the simulation of brain tissue, the goal of our investigation is to study the performance properties related to the mathematical modeling of neurons, and not implementation and hardware details such as the most efficient random number generation strategy. Despite excluding some parts of the simulation algorithm, our analysis still maintains a lot of relevance with regards to the overall simulation performance. Indeed, in both G-based and I-based models, it was found that computational and communication kernels often constitute more than 90% of the total simulation runtimes (Kumbhar et al. 2019a; Ewart et al. 2015; Schenck et al. 2014; Peyser and Schenck 2015). From the modeling point of view, an important aspect that we have neglected in this analysis is synaptic plasticity. A large portion of research questions that require brain tissue simulations involve learning and synaptic plasticity, so this represents an important extension to our analysis. However, in this work we decided to concentrate on the inference part of brain tissue simulations because the diversity and complexity of plasticity models warrants a separate analysis. However, we have shown that we do not expect synaptic plasticity to significantly change our analysis. In the future, given that the performance modeling infrastructure is already in place, we believe that the addition of plasticity for a more detailed analysis would not be a technical challenge, although it would considerably complexify the resulting analysis. Even though we already considered potential hardware improvements in our analysis, it would be interesting to extend this study to include hardware with different a design space such as the non-overlapping caches of AMD CPUs (Hager 2017) or massive SIMD parallelism of GPGPUs (Knight and Nowotny 2018). Finally, the methods developed in this paper can be extended to different simulation approaches ranging from different communication strategies (Kozloski and Wagner 2011; Ananthanarayanan and Modha 2007) up to fully asynchronous executions (Magalhães and Schürmann 2019).

### Performance Modeling is Required to Enable Next-Generation High Performance Brain Tissue Simulations

Our analysis shows that, if future iterations of general-purpose hardware architectures maintain the same balance as the current state-of-the-art, it will be very difficult to achieve fast, large scale simulations of brain tissue. Even if hardware peak performance were to improve significantly over the next years, the required speedup could only be achieved via specifically targeted advancements and under very restrictive simulation and model configurations. To support the next generation of brain tissue simulations, the community must therefore focus on the design of dedicated hardware.

In this work we have shown that different modeling choices and abstractions lead to a large diversity in the performance landscape of cellular-level simulations, in spite of being representations of the same biological phenomenon, thus making the task of designing the hardware and software necessary to achieve high performance simulations extremely difficult. The level of diversity means that design tradeoffs will inevitably require very restrictive decisions about the scale, model and configuration of the target simulation, such as e.g. (Navaridas et al. 2012; Knight and Nowotny 2018). For example, in the context of accelerating small networks of neurons up to real-time, we predict that I-based models would benefit from architectures with high memory level parallelism and a simple cache hierarchy, while for G-based models the cache bandwidth as well as the throughput of CPU arithmetic operations must be improved concurrently in order observe the desired speedup. Additionally, simulation parameters can influence the performance profile in a tangible way. In the case of the Simplified model, for example, simulations are on average bounded by memory bandwidth and network latency at the current value of the minimum network delay, but the profile would change drastically if the minimum network delay were increased to the same value of the Brunel model, with memory bandwidth losing importance in favour of cache and CPU throughput. Even in ideal cases where the *in silico* experiment falls perfectly within the design space of the hardware and software being used, simulation dynamics outside of the control of the modeler such as firing frequency can rapidly push the performance of simulations towards suboptimal regimes.

## Conclusions

We have demonstrated the diversity and complexity of the performance landscape of brain tissue simulations, using an analytical performance model that combines algorithm definitions and hardware specifications as an exploration tool. Thanks to the analytical modeling tools developed by us, we are able to gain insight on intrinsic model properties at the root of the observed performance variability across models, and to pinpoint hardware and software culprits for performance bottlenecks in brain tissue simulations. We conclude that, even though state-of-the-art published simulations are currently greatly benefitting from general purpose computing, it is likely that reaching fast, brain-scale simulations on general purpose HPC platforms will not be feasible in the near future. In order to achieve this goal, computational neuroscience modelers must cooperate with software developers and hardware designers. Ultimately, we stress that performance modeling represents a powerful tool to enable communication between these communities We believe that our work embodies a concrete step in defining and understanding key performance properties of a wide variety of *in silico* models, necessary to enable the next generation of brain tissue simulations.

## Resource Sharing Statement

All the information about the *in silico* models was obtained directly from the corresponding publications and is publicly available.

## Electronic supplementary material

Below is the link to the electronic supplementary material.

(PDF 2.88 MB)
