# Supplementary material for: Understanding Computational Costs of Cellular-Level Brain Tissue Simulations Through Analytical Performance Models
Source: Neuroinformatics. 2020 Feb 13;18(3):407–28. doi: 10.1007/s12021-019-09451-w (PMC7338826; doi:10.1007/s12021-019-09451-w)
Supplement: Supplementary file 1 — (PDF 2.88 MB) [file 12021_2019_9451_MOESM1_ESM.pdf]

## S1 Performance modeling methods

This section contains supplementary materials concerning the performance modeling methods employed in this paper.

### S1.1 Single-node ECM performance model

The Execution-Cache-Memory performance model is a *grey-box* model developed by the HPC group at the university of Erlangen. It uses a mixed approach combining an analytic formulation with some phenomenological input, and outputs a runtime prediction at the granularity of individual clock cycles. The ECM model was first introduced by Treibig and Hager (2010) and successively refined and validated on modern Intel and AMD multicore architectures (Hofmann et al. 2017, 2018; Stengel et al. 2015). A recent review provides a clean and detailed description by abstracting, formalizing and recasting it as a universal modelling approach based on a strict differentiation between application and machine models (Hofmann et al. 2019).

In the ECM model, one must first define several contributions to the runtime of a given loop. The notation of these contributions is as follows:

- $T_{OL}$: the in-core execution time assuming data is already loaded in registers;
- $T_{nOL}$: the time needed to load data into registers from the L1 cache;
- $T_{L1L2}, T_{L2L3}$: the data traffic time between caches;
- $T_{L3Mem}$: the read-only data traffic time from main memory to L2, and the write-only data traffic time from L3 to main memory;
- $T_{core}$: the total time spent in the execution of CPU instructions, assumed to overlap perfectly with data traffic;
- $T_{data}$: total time spent in data traffic, typically as a sum of individual data traffic contributions on Intel architecture.

To estimate $T_{core}$ one typically assumes that all instructions occur at maximum throughput, even though in this work we are sometimes forced to extend the model by discarding this assumption to obtain more accurate results. Code analysis tools such as the Intel Architecture Code Analyzer (IACA) (Intel 2017) or the experimental Open Source Code Analyzer (OSACA) (Laukemann et al. 2018) can provide some non-analytical input to the model, allowing to greatly increase its prediction accuracy at the cost of losing some interpretability.

To estimate $T_{data}$ one needs to provide two kinds of information: the contributions to the runtime from individual caches as well as a formula to combine them. For individual caches we must find the memory traffic, a difficult task that can be affected by the following aspects:

- total memory requirements of the kernel;
- cache reuse or blocking;
- cache associativity;
- victim caching;
- cache replacement policy.

For the relatively simple case of a kernel with no reuse, requiring a data traffic of $b$, denoting the L1L2 bandwidth with $BW_{L1L2}$ we compute the contribution:

$$T_{L1L2} = \frac{b}{BW_{L1L2}}. \tag{S1}$$

To combine the individual caches' contributions into $T_{data}$, on all recent Intel server microarchitectures, the best accuracy in predictions is obtained assuming that there is no temporal overlap between any cache transfers (Hager and Wellein 2016). Thus the formula to compute $T_{data}$, assuming the dataset must be fetched from main memory, is

$$T_{data}^{Mem} = T_{nOL} + T_{L1L2} + T_{L2L3} + T_{L3Mem}. \tag{S2}$$

Assuming there is no overlap between caches, the predictions for data originating in different levels of the cache hierarchy are defined using eq. (1) as:

$$\begin{aligned} T_{ECM}^{L1} &= max\left(T_{OL}, T_{nOL}\right), \\ T_{ECM}^{L2} &= max\left(T_{OL}, T_{nOL} + T_{L1L2}\right), \\ T_{ECM}^{L3} &= max\left(T_{OL}, T_{nOL} + T_{L1L2} + T_{L2L3}\right), \\ T_{ECM}^{Mem} &= max\left(T_{OL}, T_{nOL} + T_{L1L2} + T_{L2L3} + T_{L3Mem}\right). \end{aligned} \tag{S3}$$

To make predictions on the parallel runtime (in a shared memory configuration), an additional assumption is required. The standard ECM model assumes that performance scales linearly with the number of threads, until a bottleneck from a shared serial resource is used, typically the memory interface (Hofmann et al. 2015). Thus starting from the definition of an amount $W$ of *work* done – e.g. one fully processed Cache Line (CL) worth of data – we can define the single thread performance as

$$P(1) = \frac{W}{T}, \tag{S4}$$

where $T$ is the runtime required to complete the amount of work $W$ and can be obtained from eq. (1). From the assumption that performance scales linearly until a bottleneck is reached, e.g. $P_{BW}$ determined by the memory bandwidth, we can easily obtain the formula for shared memory parallelism using $n_t$ threads

$$P(n_t) = min\left(n_t P(1), P_{BW}\right). \tag{S5}$$

The ECM model allows to compute the *saturation point*, defined as the number of threads at which the serial memory bottleneck is saturated and dominates performance. Once this point is reached, the kernel's performance cannot be improved by increasing the number of threads. It is possible to

explicitly compute the number of threads at which saturation occurs, using the formula:

$$n_{satur} = \left\lceil \frac{T^{Mem}}{T_{L3Mem}} \right\rceil . \quad \text{(S6)}$$

Another quantity that we are often interested in computing is bandwidth utilization, defined based on the kernels's data traffic requirements $b$ as:

$$\begin{aligned} BW_{util} &= \frac{BW_{expressed}}{BW_{Mem}}, \\ BW_{expressed} &= \frac{b}{T^{Mem}} [\text{GB/s}]. \end{aligned} \quad \text{(S7)}$$

To put the emphasis on the hardware bottlenecks identified by the ECM model, we can "invert" the runtime prediction formulas to obtain a description in terms of hardware contributions. In the serial case, hardware contributions are simply defined based on the regular ECM dimensions. We define the contributions as follows:

$$\begin{aligned} T_{core} &= \begin{cases} T_{OL} & T_{OL} \geq T_{data} \\ 0 & \text{otherwise} \end{cases} \\ T_{caches} &= \begin{cases} 0 & T_{OL} \geq T_{data} \\ T_{nOL} + T_{L1L2} + T_{L2L3} & \text{otherwise} \end{cases} \\ T_{DRAM} &= \begin{cases} 0 & T_{OL} \geq T_{data} \\ T_{L3Mem} & \text{otherwise} \end{cases} \end{aligned} \quad \text{(S8)}$$

In the parallel case, however, special care must be taken to distinguish scalable and non-scalable contributions. Scalable contributions are logically improved by adding more threads, and usually correspond to physical hardware that is replicated for each thread. The scalable contributions are $T_{core}$ and $T_{caches}$, while the non-scalable contribution is $T_{DRAM}$. In a parallel execution using $n_t$ threads, we define the hardware contributions as follows: non-scalable contributions remain unchanged from the serial execution; scalable contributions are scaled by $\frac{1}{n_t}$ until saturation of the memory bandwidth, then set to 0.

*Reference architecture: Intel Skylake-X* The methodology proposed in this paper is general, but to obtain concrete performance predictions and bottleneck analysis we are required to focus on a target architecture. To uphold a satisfactory level of relevance for the high performance computing community as well as generalizability to future architectures, we picked a modern, general-purpose Intel server architecture, a Intel Skylake (SKX) Intel(R) Xeon(R) Gold 6140 with AVX512 vectorization and Sub-NUMA clustering turned off. The most relevant hardware characteristics required by the performance model are summarized in Table S3, but we refer to (Cremonesi et al. 2019) for the full details. We obtained these values either directly from the vendor's spec sheets, by custom-designed benchmarks or from the reference tables in (Fog 2017).

**Table S3** Hardware characteristics of reference architecture SKX AVX512.

| | value | unit |
|---|---|---|
| CPU freq | 2.3 | GHz |
| Peak DP performance | 1324.8 | Gflop/s |
| Mem BW | 105 | GB/s |
| L1-L2 BW per core | 64 | B/cy |
| L2-L3 BW per core | $2 \times 16$ | B/cy |
| vector `exp()` throughput | 1.5 | cy/scalar iter |
| scalar `exp()` latency | 22.2 | cy/scalar iter |

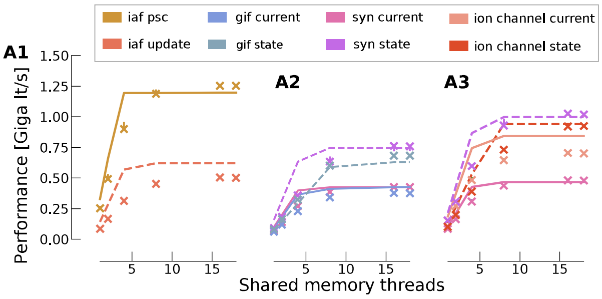


**Fig. S10** Validation of performance model applied to clock-driven kernels of *in silico* models. Performance is measured as Giga iterations per wallclock second for a single instance of the kernel. The reference architecture is SKX AVX512. Lines represent our predictions using the ECM model, while markers represent median benchmark measurements. Error bars represent the 25%-75% percentiles, but variability is so low that they are often hidden. To improve readability we used dashed lines for state update kernels and solid lines for current kernels. All benchmarks were designed with big enough datasets to ensure data was always coming from DRAM. **A,B,C** Clock-driven kernels of the Brunel, Simplified and Reconstructed model respectively.

*ECM model of clock-driven kernels* We computed the ECM model for all the relevant kernels constituting the simulation workflow presented in Fig 2. We present the results in Table S4. The units for all runtime predictions are cycles per scalar iteration, denoted as [cy], while the units for performance are Giga-scalar iterations per second, denoted by [Giga/s].

*Validation of clock-driven kernels* To validate our predictions we benchmarked and measured the serial and parallel runtime of the individual kernels in a simulation that is representative of a typical workload. Due to overheads it was impossible to design benchmarks for the L2 and L3 caches for the point neuron kernels described above. Therefore all the benchmarks presented here have a sufficiently large dataset to only fit in DRAM. For all *in silico* models we validated our predictions for the serial execution as well as for shared memory scaling. ealidation results for the Brunel, Simplified and Reconstructed model are presented in Figure S10 and Table S5. We scale all our performance measurements by the reported average frequency during the execution of that kernel.

**Table S4** ECM model of clock-driven kernels of *in silico* models and experiments. For simplicity, we only report the model based on the AVX512 vectorisation. The ECM contributions and predictions are reported in cy per scalar iteration. Horizontal lines distinguish the three *in silico* models: Brunel (B), Simplified (S), Reconstructed (R).

| | kernel name | $T_{OL}$ | $T_{nOL}$ | $T_{L1L2}$ | $T_{L2L3}$ | $T_{Mem}$ | $T_{ECM}^{L1}$ | $T_{ECM}^{L2}$ | $T_{ECM}^{L3}$ | $T_{ECM}^{Mem}$ |
|---|---|---|---|---|---|---|---|---|---|---|
| B | iaf update | 2.41 | 1.75 | 2.62 | 8.00 | 3.68 | 2.41 | 4.38 | 12.38 | 16.05 |
| | iaf psc | 0.62 | 0.38 | 1.25 | 3.00 | 1.75 | 0.62 | 1.62 | 4.62 | 6.38 |
| S | synapse current | 7.44 | 3.50 | 3.51 | 9.03 | 4.92 | 7.44 | 7.44 | 16.03 | 20.95 |
| | gif current | 9.88 | 3.38 | 3.75 | 11.00 | 5.26 | 9.88 | 9.88 | 18.12 | 23.38 |
| | synapse state | 13.31 | 2.62 | 2.00 | 5.50 | 2.80 | 13.31 | 13.31 | 13.31 | 13.31 |
| | gif state | 28.50 | 6.25 | 2.38 | 6.50 | 3.33 | 28.50 | 28.50 | 28.50 | 28.50 |
| R | synapse current | 7.20 | 3.50 | 3.21 | 8.33 | 4.50 | 7.20 | 7.20 | 15.04 | 19.54 |
| | ion channel current | 4.68 | 2.56 | 1.92 | 5.07 | 2.70 | 4.68 | 4.68 | 9.55 | 12.25 |
| | linear algebra | 8.10 | 6.00 | 1.40 | 4.00 | 1.90 | 8.10 | 8.10 | 11.40 | 13.30 |
| | synapse state | 9.70 | 1.70 | 1.50 | 4.00 | 2.10 | 9.70 | 9.70 | 9.70 | 9.70 |
| | ion channel state | 15.82 | 2.16 | 1.59 | 3.56 | 2.24 | 15.82 | 15.82 | 15.82 | 15.82 |

**Table S5** Validation of ECM performance model for clock-driven kernels from all *in silico* models and experiments. Validation conducted using the SKX AVX512 reference architecture. The parallel column refers to full-chip shared memory parallelism (18 threads). Measurements are shown as median values ± interquartile range from a dataset of 10 independent benchmark executions. Runtime measurements and predictions are reported in cy per scalar iteration. Horizontal lines distinguish the three *in silico* models: Brunel (B), Simplified (S), Reconstructed (R).

| | | serial | | parallel | | memory volume | |
|---|---|---|---|---|---|---|---|
| | kernel name | pred [cy] | meas [cy] | pred [cy] | meas [cy] | pred [B] | meas [B] |
| B | iaf update | 16.1 | 26.4±0.9 | 3.7 | 4.5±0.0 | 168 | 193.6 ± 0.9 |
| | iaf psc | 6.4 | 8.2±0.6 | 1.8 | 1.7±0.0 | 80 | 76.2 ± 0.8 |
| S | synapse current | 21.0 | 28.9±1.8 | 4.9 | 4.9±0.1 | 224 | 225 ± 1.7 |
| | gif current | 23.4 | 33.7±1.7 | 5.3 | 6.0±0.2 | 232 | 237.1 ± 12.7 |
| | synapse state | 13.3 | 21.1±0.4 | 2.8 | 2.8±0.1 | 128 | 127.7 ± 0.1 |
| | gif state | 28.5 | 25.4±0.0 | 3.3 | 3.1±0.0 | 144 | 123 ± 0.6 |
| R | syn current | 19.5 | 24.6±1.5 | 4.5 | 4.4±0.1 | 205 | 207.1 ± 2.1 |
| | ion channel current | 12.2 | 15.2±0.3 | 2.7 | 3.3±0.1 | 123 | 120.3 ± 11.0 |
| | linear algebra | 13.3 | 18.8±5.3 | 1.9 | 2.2±0.2 | 88 | 90.7 ± 4.2 |
| | syn state | 9.7 | 13.8±0.2 | 2.1 | 2.0±0.0 | 96 | 94.3 ± 1.3 |
| | ion channel state | 15.8 | 20.6±0.1 | 2.2 | 2.3±0.0 | 100 | 99.9 ± 2.0 |

In the serial case, the prediction errors are all within 20-30% of the measured runtime. Obtaining more accurate predictions is challenging, and the reasons for this can vary across kernels. For G-based state kernels a long critical path in the loop kernel code could be weakening the accuracy of our predictions due to a failure of the full throughput assumption, while errors in the ion channel current predictions could be imputable to memory traffic overhead due to indirect memory addressing. While it is still possible to obtain reasonably accurate predictions for the state kernels, it must be noted that ultimately it is extremely hard to predict the dynamic behaviour of the out-of-order engine in a complex, modern architecture. Finally, given that most of the predictions in this case are optimistic, it is reasonable to assume that performance limiting factors such as dynamic CPU throttling, as well as intrinsic factors such as critical paths and instruction latencies, could be impacting the performance negatively.

*Validation of the event-driven spike delivery kernel* This kernel is characterised by erratic memory accesses, because the order of activation of synapses is unpredictable. We always consider the worst possible case in which every spike to be delivered could not be cached and thus must come from main memory. This approximation has a strong impact on our estimates of the memory traffic and the scalability of the spike delivery kernel, notably in the strong scaling scenario, but we believe it represents a valid heuristic because of the very low activation of individual synapses. Indeed, given that physiological values of the firing frequency lie around $1Hz$, this means that each synapse would receive an event roughly once every 40000 time iterations, such that caches implementing a LRU policy would most likely have gotten rid of the corresponding cache line by then. Due to the erratic access, one is tempted to speculate that memory latency will be a dominant factor in the performance of this kernel. Upon deeper analysis, we find that spike delivery kernels are indeed affected by the latency of the memory system, albeit

not dominated by it. The reason is that while the CPU is handling the delivery of one spike, it has potentially access to the information about the index of the next spikes to be handled, since it already read several values from the spike events array. Thus the CPU is in principle able to schedule as many memory accesses in advance as its queue of outstanding memory requests allows it to, partially hiding the latency of processing individual spikes. This is different from the classic purely latency bound kernels in which the CPU is only allowed to begin a loop iteration after the previous one is fully completed. On the other hand, all the requests for data are non-contiguous and therefore we expect that none of the pipelining and prefetching techniques are very effective in hiding the latency of fetching the data.

We assume that a full cache line of data needs to be brought in from memory *for every data access*, since the unpredictable order of activation of synapses renders data prefetching and data blocking largely ineffective. Note that because of write-allocate, every write operation counts as two accesses. To build a performance model, we are now tasked with figuring out which memory accesses should be considered non-contiguous and thus represent a potential issue for performance. For non-contiguous memory accesses, the predicted memory traffic is quite large because we assume that a full cache line (64 B) must pulled from the memory even though only a single double-precision variable (i.e. 8 B) would be required. We consider that only accesses to synapse-specific data are non-contiguous and thus require a full cache line (64 B) of data to be transferred for every memory request. In our implementation, the G-based kernel requires 22 non-contiguous data accesses for a total of 1408 B per iteration, and the I-based kernel a meagre 2 accesses for a total of 128 B per iteration. In benchmarks, we measure a memory traffic of $1396.9 \pm 2.2$ B per iteration for the G-based kernel, and $149.3 \pm 11.9$ B for the I-based kernel, thus lending high credibility to this assumption. Figure S11B shows a validation of the estimated memory traffic against realistic benchmarks.

Estimating the runtime proves to be a very challenging task. We find that the naïve approach of multiplying the DRAM latency by the number of non-contiguous accesses yields very pessimistic predictions. This can be attributed to the fact that, since spikes are independent, it is not necessary to wait until one spike has been processed before issuing request for the data of the next spike. It thus seems that the spike delivery kernel's performance is determined by the number of concurrent, independent data requests that can be handled by the processor and memory. This is different from the classical purely latency bound kernels in which the CPU is only allowed to begin a loop iteration after the previous one is fully completed. The number of independent memory requests that can be handled concurrently is known as memory level parallelism (MLP) and allows to

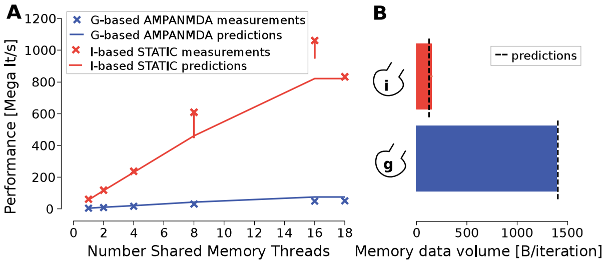


**Fig. S11** Validation of the spike delivery kernel. **A** Performance predictions (dashed lines) and benchmark measurements (X markers). Performance is measured in Mega-delivered spikes per wallclock second. Error bars represent the 25% and 75% percentiles. **B** Validation of memory traffic per delivered spike. Bars represent measurements, dashed lines represent our predictions based on the worst-case scenario. Memory traffic is measured in B per delivered spike.

mitigate the performance impact of memory latency by allowing multiple accesses in parallel (Levinthal 2014). Using an appropriate benchmark (see e.g. Lemire 2018) for an example we measured an adjusted memory latency of roughly 20 cy per memory access on our reference architecture. For shared memory parallelism, we assume that performance scales linearly with the number of threads until the bottleneck of memory bandwidth is reached.

In the case of the G-based model, the procedure above leads us to a single-thread prediction of $22 \times 20 = 440$ cy per delivered spike, to be compared to the benchmark measurement of $571.6 \pm 14.9$ cy, which represents roughly a 23% error. For multiple threads, we assume that the performance scales linearly with the number of threads until the bottleneck of memory bandwidth is exhausted. At the maximum number of threads, this amounts to a predicted runtime of 30.8 cy per delivered spike, against benchmark measurements of $45.0 \pm 0.1$ cy, i.e. a 31% error. For the I-based model, we predict a serial runtime of 40 cy and measure 38.0 $\pm$ 1.8 cy, giving a small error margin of 5%, while at maximum thread we predict a runtime of 2.8 cy and measure $2.8 \pm 0.04$. The memory traffic estimates and the runtime estimates are all within an acceptable margin of error for both models. Given the complexity introduced by the out-of-order execution and memory access scheduling, we deem these predictions quite satisfactory. In Fig.S11 we present the predicted and measured performance and memory traffic for the spike delivery kernel

## S1.2 Interprocess communication LogGP performance model

The LogGP model is part of a family of analytic performance models developed initially at Berkeley, was adapted and extended by researchers from other centres. Historically, the first model in this family was LogP (Culler et al. 1993).

In the LogP model all complex MPI operations are defined on the basis of point-to-point communication, i.e. the cost of sending a message from one compute node to another within the same network. LogP was developed with a focus solely on short (single Byte) messages, but it quickly became clear that the accuracy of its predictions degraded in the case of long messages. Therefore the LogGP model (Alexandrov et al. 1997; Hoefler et al. 2009) was developed to overcome this problem. In the LogGP model, the cost of sending a single message of size $m$ B is given by the analytic formula

$$T_{pt2pt} = L + 2o + G(m-1), \tag{S9}$$

where

- $L$ is the network latency;
- $o$ is the overhead from non-network operations;
- $g$ is the inverse of the injection rate;
- $G$ is the inverse of the network bandwidth;
- $P$ is the number of processes involved in the communication.

Note that $g$ does not appear above because a single message is considered, while $g$ represents the delay that must occur between two consecutive sends of a message.

*LogGP model on Infiniband EDR with HPE-MPI* While the performance modelling tools considered here can generalize well to several types of architectures (Hoefler et al. 2009), to validate our performance predictions we restrict our focus to a representative example of an HPC network architecture: a vendor (HPE) MPI implementation based on MPT 2.16 and the MPI 3.0 standard, over an Infiniband EDR 100 GB/s fabric. While it would be possible to take the nominal vendor values for the hardware parameters such as $L, g, G$, it is highly advised to obtain the values of these parameters through a set of benchmarks. A low-overhead method to compute all the necessary parameters has been proposed (Hoefler et al. 2007a), and ultimately led to the development of the Netgauge tool (Hoefler et al. 2007b). The model parameters can be obtained once and for all, and after that the LogGP model does not require any additional benchmarking efforts. We used the Netgauge v2.4.6 tool introduced in (Hoefler et al. 2007a) to make a first assessment of the LogGP parameters, and the parameters reported in Table S6.

The LogGP framework allows us to directly model the latency of point-to-point communication using the formula $(L+2o_i)+(G+2o_s)m$, where $m$ is the message size. However, additional work is required to model collective communication because different algorithms can be used to disseminate the messages across the network. For the Allgather operation there are several implementations available, and the decision of which one to use can be a complex function of the static network characteristics such as the topology as well as dynamic specifications such as the message size and number of ranks involved (Thakur et al. 2005). In this work we consider only the ring algorithm, because it is the mosts commonly used (Thakur et al. 2005) (especially for large message sizes) and we wish to keep our analsys simple. Therefore, the total latency to perform an Allgather operation among $P$ parallel ranks with a total message size of $m$ is:

$$T_{Allgather} = (P-1)(L+2o_i) + \frac{P-1}{P}(G+2o_s)m. \tag{S10}$$

**Table S6** LogGP parameters.

| | small sizes | large sizes | unit |
|---|---|---|---|
| $L$ | 1.54 | 1.54 | $\mu$s |
| $o_i$ | 0.133 | 0.0249 | $\mu$s |
| $o_s$ | $4.59 \times 10^{-5}$ | $6.48 \times 10^{-5}$ | $\mu$s/B |
| $g$ | 0.526 | 6.12 | $\mu$s |
| $G$ | $1.42 \times 10^{-4}$ | $2.07 \times 10^{-4}$ | $\mu$s/B |

*Validation and inference* The original CoreNEURON implementation of the spike exchange algorithm uses an MPI custom datatype to represent a spike as an aggregate of a double-precision variable representing the time of spike and an integer variable representing the ID of the source neuron. This implementation, however, can turn out to be unsatisfactory in terms of performance, in particular when one tries to predict the runtime of a collective communication, because hidden data shuffling and copy operations can take place (Carpen-Amarie et al. 2017). To address the performance issues deriving from custom datatypes, we reimplemented the spike exchange operation to perform two consecutive MPI_allgatherv operations: the first on the array of timings and the second on the array of source neuron IDs. To measure the latency of the communication itself, we executed a simulation where we artificially filled every communication buffer on each distributed rank with the same number of spikes, and measured the communication time per rank.

We tested our model in the actual simulation environment, by artificially contriving neurons to fire a predetermined amount of spikes at each time interval. Results are presented in Figure S12. Computation of the LogGP predictions in this case requires careful treatment of the message size. We identify three regions of interest: the first one where the total message size is so small that the condition $(m < 65P)$ is satisfied for communication of both the neuron IDs (integers) and the spike times (doubles); a second region where the performance penalty is valid for the communication of the spike times, but not the neuron IDs; a third region where the performance penalty applies to both communication phases. Our model seems to have small optimistic bias, but overall it always falls within a 10% error region.

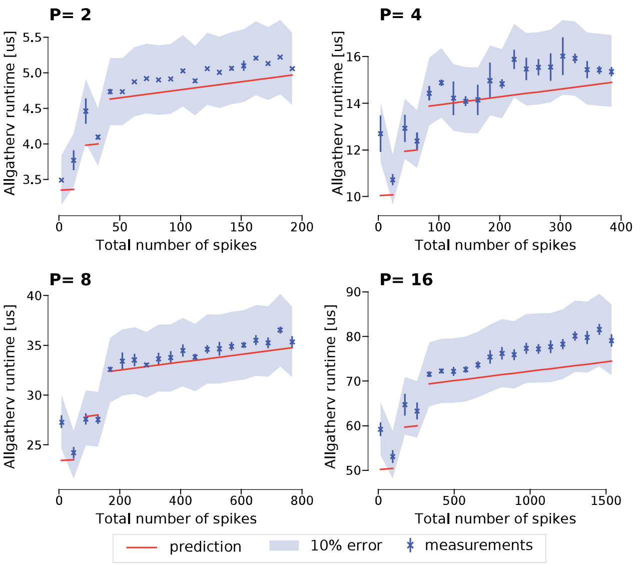


**Fig. S12** Validation of LogGP model for interprocess spike exchange in the simulation environment. Weak scaling of a CoreNEURON simulation where individual neurons are contrived to emit a predetermined number of spikes per timestep. All neurons emit the same number of spikes. Measurements are obtained by summing the contributions from communicating the IDs of spiking neurons and the time of spiking $T_{comm,ID} + T_{comm,t}$, while predictions are split in three regions according to message size as described in the text. Red lines represent the model's predictions, while blue markers represent the measured latency averaged across parallel ranks. Error bars represent the minimum and maximum latency across parallel ranks. The shaded area represents a 10% error w.r.t measurements.

## S2 Effect of model parameters on simulation performance

Parameters of the *in silico* models have an important, yet often difficult to explain, impact on performance. We test the impact of the firing frequency, minimum network delay and fan in using our performance model, and present the results in this section. We neglect the timestep because it has a generally straightforward relationship with performance, and was omitted for brevity.

*Firing frequency's differential effect on communication and computation* Firing frequency is commonly cited as one of the most impactful parameters on simulation performance (Yavuz et al. 2016). In this work we consider it a parameter even though it usually cannot be explicitly set by the user, and is instead an emerging property of the simulation. Fig.S13A shows the predictions of our performance model for the three *in silico* models, for values of the firing frequency in a physiological range. To take into account the obvious fact that the total number of neurons and distributed ranks can introduce a large variability in our predictions, we randomly generate 1000 value pairs of [number of neurons, number of distributed ranks] and plot the median predicted performance, additionally broken down into its two components of inter-node communication and on-node computation. For the number of neurons we consider values in the range $[1, 10^8]$ while for the number of ranks we consider values in the range $[1, 10^3]$; furthermore, we discard the few configurations for which the number of neurons was randomly chosen to be smaller than the number of ranks, as that would imply the splitting of neurons. We generate random numbers of neurons and ranks following a log-uniform distribution, with the effect that all orders of magnitude are equally likely, thus introducing a very large variability. Firing frequency has an effect on communication by changing the size of the spike message as well as on computation by changing the amount of events that must be integrated by neurons. In Fig.S13A it is noticeable that there exists a threshold frequency below which $f$ does not affect performance, but once this threshold is passed firing frequency becomes a primary factor, inducing a linear, almost unit-slope degradation in performance. This effect is clearly visible in the Simplified model, and even more so in the Brunel models. For the Reconstructed model this threshold value exists, but is so large that we can safely assume that, in the median case, firing frequency has no effect on performance. To investigate the reasons for performance degradation we look at the breakdown of relative importance of different hardware features as a function of the firing frequency, plotted in Fig.9A. Similarly to before, we randomly generate 1000 couples of [number of neurons, number of distributed ranks] but we plot the mean relative importance instead of the median to keep the total constantly equal to 100%. Our analysis shows an interesting behaviour: as the firing frequency becomes larger, the relative pressure on the memory bandwidth (and eventually the network bandwidth) becomes larger, while the relative pressure on the network latency becomes smaller. So not only there is more computation to be done as firing frequency gets larger, but also the mix of hardware bottlenecks changes. This behaviour was observed empirically not only on general-purpose CPUs (Zenke and Gerstner 2014) but also on GPUs which are additionally more susceptible to dynamic load balancing because of the extremely large number of parallel cores (Yavuz et al. 2016) We remark that the large variability in the performance predictions in Fig.S13A,B,C can be at least partially explained by our choice of sampling strategy as mentioned above.

*Minimum network delay affects the relative importance of hardware features* Another parameter of interest is the minimum network delay, denoted $\delta_{min}$. In terms of communication, the minimum network delay affects the number of times that global communication must happen to simulate one second of biological time, although it does not affect the total number of spikes communicated. In terms of computation, assuming that the loop ordering strategy to minimise pressure on the memory bandwidth is employed then

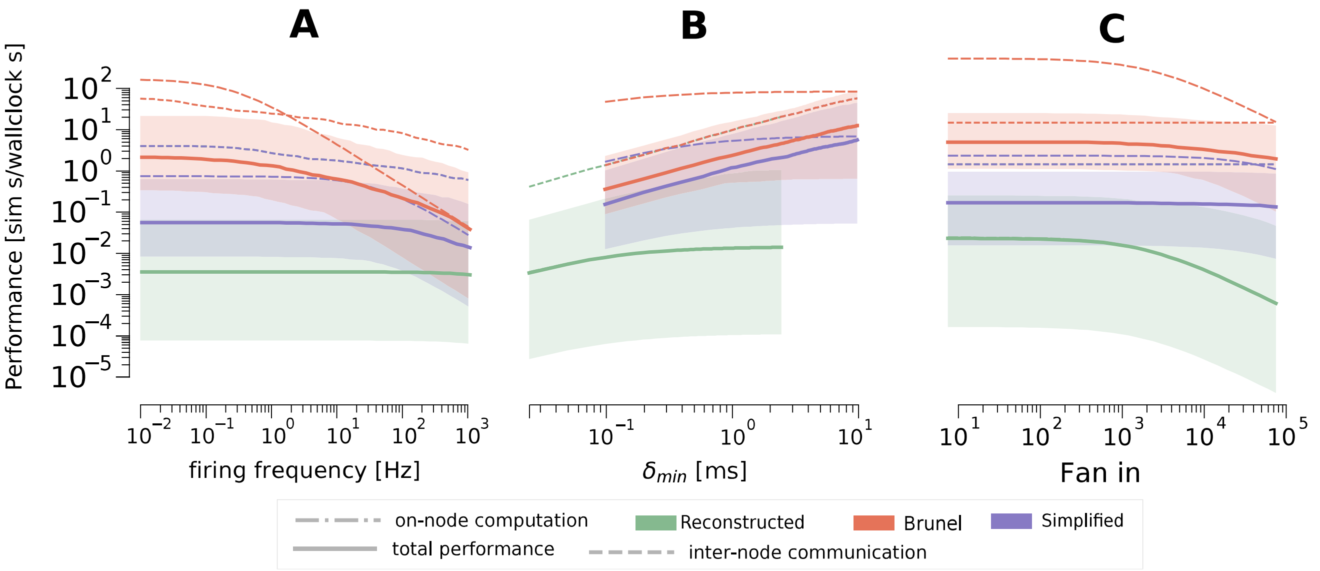


**Fig. S13** Effect of model parameters on performance. **A** Effect of firing frequency on the performance of distributed simulations. We plot the median performance over 1000 randomly generated simulation samples defined by number of neurons and number of distributed ranks. The shaded area surrounding the total performance represents the 25th and 75th percentiles. **B** Effect of $\delta_{\text{min}}$ on the performance of distributed simulations. The range of acceptable values for $\delta_{\text{min}}$ changes across different *in silico* models because they were computed as multiples of the model's timestep. **C** Effect of fan in $K$ on the performance of distributed simulations.

the minimum network delay affects the number of time iterations in which data locality can be exploited. Fig.S13B shows the predictions of our performance model for the three *in silico* models, for different values of the minimum network delay. Since this delay can only be an integer multiple of the timestep, we exploit the concept of coupling ratio to define a range of plausible minimum delay values by setting a range of values for the coupling ratio and obtaining the corresponding $\delta_{\text{min}}$ by multiplication with $\Delta t$. By looking at the breakdown of performance, we see that larger minimum network delay values improve the performance of inter-node communication but also, somewhat surprisingly, on-node computation. However, while the communication performance seems to improve indefinitely, the improvement of on-node computation saturates quite quickly. For the point neuron models, within the range of minimum network delay values considered here, there is a transition from a regime dominated by communication to one dominated by computation, while the Reconstructed model is dominated by computation for all minimum network delay values. To investigate the reasons for changes in performance, we plot the breakdown of relative importance of different hardware features in Fig.9B. In the case of G-based models, larger minimum network delay values correspond to decreased pressure on the memory bandwidth and network latency, and larger pressure on more scalable hardware features such as CPU instruction throughput and caches throughput. This points to the fact that simulations based on G-based models with a large minimum network delay could strongly benefit from shared-memory parallelism. On the other hand, a larger minimum network delay in the I-based model results in decreased pressure on the network latency, but a higher pressure on memory bandwidth.

*Large fan in can be advantageous for performance of point neuron models, but has almost no effect on Reconstructed model* Finally, we examine the effect of fan in, defined as the average number of incoming connections per neuron and denoted by $K$. This parameter has subtle effects on performance that are difficult to analyse. For G-based models, a larger fan in technically means more synapses to simulate thus an expected degradation of performance. Additionally, for all models a larger $K$ determines an increase in event-driven computation, thus once again an expected degradation of performance. We confirm this with the analysis in Fig.S13C showing that $K$ does not affect communication but has a very strong effect on the Reconstructed model. Unexpectedly, fan in seems to only marginally affect the performance of the Simplified model, in spite of it being a G-based model too. This can be easily explained by the fixed number of synaptic instances in this model (28 excitatory and 8 inhibitory (Rössert et al. 2016)), such that much like the I-based Brunel model, ultimately the fan in affects only the average number of events a neuron must integrate within a certain time period. Another important point should be made about the effect of fan in, because changing the number of connections of a neuron has an impact on the *in silico* model's memory requirements. Fig.9C shows the ratio of neurons that can fit in 1 GB of memory, according to our performance model, as a function of

fan in. In a strong scaling scenario, this information sheds new light on the conclusions above, because if only $\frac{1}{x}$ neurons fit in 1 GB, this can result potentially in a $x$-fold increase in performance from parallelism (disregarding potential communication bottlenecks). Therefore for the Brunel and Simplified model it can be advantageous to have a large number of incoming connections per neuron, because the performance price paid is more than compensated by the required increase in parallelism. Conversely, in the Reconstructed model, these two effects appear to balance out almost evenly.

For very large scale simulations, another subtle effect of fan in is represented by the size of the connection table, an issue that was raised and investigated in (Kunkel et al. 2014). The connections table of a given rank contains all the global identifiers of presynaptic neurons that are relevant for at least one neuron in that rank. The size of the connection table depends, among other things, on $K$ as well as the total number of neurons and number of distributed ranks (Kunkel et al. 2014). In Fig.9D we plot the values of the expected total size of the connection table on the (total number of neurons, number of distributed ranks)-plane as a contour plot, highlighting the contours corresponding to a total size of 1kB, 1MB and 1GB. Although in some *in silico* models connectivity may be determined by complex rules influenced by cell type and spatial locality, for simplicity we compute here the expected size of the connections table assuming uniform connection probability and random distribution of neurons across ranks. In a strong scaling scenario the size of the connections table steadily decreases as the number of ranks increases, starting from the minimum of two ranks required by a distributed simulation. This can be explained by the fact that fixing the network size and increasing the number of ranks entails that there will be less incoming connections to a given rank. In a weak scaling scenario, such as the maximum filling regime, the size of the connections table transiently increases when the number of distributed ranks is low, but at large scale reaches a constant value steady state determined only by $K$.

## Supplementary References


Carpen-Amarie A, Hunold S, Träff JL (2017) On expected and observed communication performance with mpi derived datatypes. Parallel Computing 69:98–117, doi:10.1016/j.parco.2017.08.006

Fog A (2017) Instruction tables: Lists of instruction latencies, throughputs and micro-operation breakdowns for intel, amd and via cpus. technical university of denmark

Hager G, Wellein G (2016) The execution-cache-memory model, URL `https://moodle.rrze.uni-erlangen.de/pluginfile.php/12401/mod_resource/content/1/07_06_2016_ECM_Model.pdf`, seminar on efficient numerical simulation on multi- and manycore processors

Hoefler T, Lichei A, Rehm W (2007a) Low-overhead loggp parameter assessment for modern interconnection networks. In: 2007 IEEE International Parallel and Distributed Processing Symposium, IEEE, pp 1–8, doi:10.1109/ipdps.2007.370593

Hoefler T, Mehlan T, Lumsdaine A, Rehm W (2007b) Netgauge: A network performance measurement framework. In: International Conference on High Performance Computing and Communications, Springer, pp 659–671, doi:10.1007/978-3-540-75444-2_62

Laukemann J, Hammer J, Hofmann J, Hager G, Wellein G (2018) Automated instruction stream throughput prediction for Intel and AMD microarchitectures. CoRR abs/1809.00912, URL `http://arxiv.org/abs/1809.00912`, accepted for publication, `1809.00912`

Lemire D (2018) Measuring the memory-level parallelism of a system using a small c++ program? 2018-11-05 post on Daniel Lemire's blog
